# Incidence of Lyme Borreliosis in Germany: Exploring Observed Trends Over Time Using Public Surveillance Data, 2016–2020

**DOI:** 10.1089/vbz.2022.0046

**Published:** 2023-04-12

**Authors:** Jozica Skufca, Thao Mai Phuong Tran, Gordon Brestrich, Andreas Pilz, Andrew Vyse, Claudius Malerczyk, Mendwas Dzingina, Elizabeth Begier, Maxim Blum, Margarita Riera-Montes, Bradford D. Gessner, James H. Stark

**Affiliations:** ^1^ Epidemiology & Pharmacovigilance, P95, Leuven, Belgium.; ^2^ Pfizer Pharma GmbH, Berlin, Germany.; ^3^ Pfizer Global Medical Affairs, Vaccines, Vienna, Austria.; ^4^ Vaccines Medical Affairs, Pfizer Ltd., Walton Oaks, United Kingdom.; ^5^ Patient Health and Impact, Chief Business Office, Pfizer Inc, New York, New York, USA.; ^6^ Vaccine Clinical Research and Development, Pfizer Inc, Pearl River, New York, New York, USA.; ^7^ Vaccines Medical Development & Scientific/Clinical Affairs, Pfizer Inc, Collegeville, Pennsylvania, USA.

**Keywords:** Lyme borreliosis, tick-borne disease, surveillance, spatial epidemiology, incidence, Germany

## Abstract

**Background:**

Public surveillance of Lyme borreliosis (LB) occurs in 9 out of 16 federal states of Germany and remains a critical facet of disease epidemiology and trends. We describe the incidence, time trends, seasonality, and geographic distribution of LB in Germany using publicly reported surveillance data.

**Methods:**

We obtained LB cases and incidence (2016−2020) from the online platform SurvStat@RKI 2.0, maintained by the Robert Koch Institute (RKI). Data included clinically diagnosed and laboratory-confirmed LB reported by nine out of 16 federal states of Germany where LB notification is mandatory.

**Results:**

During 2016−2020, the nine federal states reported 63,940 LB cases, of which 60,570 (94.7%) were clinically diagnosed, and 3370 (5.3%) also had laboratory confirmation, with an average of 12,789 cases annually. Incidence rates were mostly stable over time. The average annual LB incidence was 37.2/100,000 person-years and varied by spatial level, ranging from 22.9 to 64.6/100,000 person-years among nine states; from 16.8 to 85.6/100,000 person-years among 19 regions; and from 2.9 to 172.8/100,000 person-years among 158 counties. Incidence was lowest among persons 20–24 years old (16.1/100,000 person-years) and highest among those 65–69 years old (60.9/100,000 person-years). Most cases were reported between June and September, with a peak in July of every year.

**Conclusion:**

The risk of LB varied substantially at the smallest geographic unit and by age group. Our results underscore the importance of presenting LB data at the most spatially granular unit and by age to allow implementation of efficient preventive interventions and reduction strategies.

## Introduction

Lyme borreliosis (LB) is an infectious, vector-borne disease caused by the spirochete *Borrelia burgdorferi sensu* lato and mainly transmitted by the Ixodes species ticks (Cardenas-de la Garza et al. 2019). Seroprevalence studies have shown that *Borrelia burgdorferi* infections are endemic in all parts of Germany (Rath et al. 1996, Dehnert et al. 2012, Wilking et al. 2015, Woudenberg et al. 2020). Several other observational studies, including surveillance data analyses, have documented the incidence in most regions of Germany (Huppertz et al. 1999, Muller et al. 2012, Lohr et al. 2015, Akmatov et al. 2021).

In Germany, LB is not a nationally notifiable disease. Each of the 16 German federal states has the authority to determine whether to conduct LB surveillance. Currently, nine states, covering 42% of the total German population, have implemented mandatory notification for the three most common LB manifestations (erythema migrans [EM], acute neuroborreliosis [LNB], and Lyme arthritis [LA]) under state-specific regulations. The nine states include the south German state of Bavaria (reporting since 2013), the east German states of Berlin, Brandenburg, Mecklenburg-Vorpommern, Saxony, Saxony-Anhalt, and Thuringia (reporting since the 1990s), and the west German states of Rhineland-Palatinate and Saarland (reporting since 2011) (Enkelmann et al. 2018).

While limited in geographic scope, existing surveillance can provide a robust source of granular, confirmed case data to explore trends over time, geographic distribution, and demographics. Accordingly, previous analyses using the surveillance system have been conducted. Initial studies using surveillance data for clinical or laboratory-diagnosed LB reported by six eastern states indicated that LB incidence increased from 17.8/100,000 person-years in 2002 to 37.3/100,000 person-years in 2006; a second study reported that incidence decreased from 34.9/100,000 person-years in 2009 to 19.5/100,000 person-years in 2012 (Fulop and Poggensee 2008, Wilking and Stark 2014). Another study of surveillance data from nine states during 2013−2017 reported an average annual incidence of 33/100,000 person-years, although no temporal trend was discernible (Enkelmann et al. 2018). Our current analysis sought to analyze recently reported LB epidemiology on a spatially granular geographical level. This could help public health stakeholders develop geographically focused prevention strategies, which may eventually include vaccines.

## Materials and Methods

### Surveillance

Laboratories or physicians reported LB cases to the local public health offices and then to the state health offices, which transferred data to the Robert Koch Institute (RKI), the national public health institute responsible for collating and reporting data. An LB case reported to the RKI was defined as a person with any of the three following criteria: clinically diagnosed EM, laboratory-confirmed LA, or laboratory-confirmed acute LNB (Robert Koch Institute 2009). Case definitions of LB are shown in Table 1. Although EM, LA, and LNB were notifiable in nine states in Germany, the available database did not report LB by manifestation.

**Table 1.** Case Definitions for Lyme Borreliosis in the Nine German States with Lyme Borreliosis Mandatory Notification

Diagnosis	Clinical case definition	Laboratory case definition
Erythema migrans	Enlarging, red or bluish-red rounded spot or multiple spots, often with central paling	NA
Acute neuroborreliosis	Presence of at least one of the following three criteria: Acute radiculoneuritis Meningitis Acute cranial nerve palsy	Acute radiculoneuritis/meningitis Detection of lymphocytic pleocytosis and positive result with at least one of the following three methods: Elevated CSF/serum antibody index to detect intrathecally formed antibodies Pathogen isolation in CSF (culture) Nucleic acid detection in CSF (*e.g.*, PCR) Acute cranial nerve palsy Positive result with at least one of the following four methods: IgG antibody detection (*e.g.*, ELISA), confirmed, for example, with Western blot or line assay (only for children and adolescents up to 18 years of age) Elevated CSF/serum antibody index to detect intrathecally formed antibodies Pathogen isolation in CSF (culture) Nucleic acid detection in CSF (*e.g.*, PCR).
Lyme arthritis	Presence of both of the following criteria: Mono- or oligoarthritis occurring for the first time (possibly intermittently) AND The exclusion of arthritis of other origins (*e.g.*, reactive arthritis and diseases of the rheumatic type).	Positive result with at least one of the following three methods: IgG antibody detection (*e.g.*, ELISA), confirmed, for example, with Western blot or line assay Pathogen isolation in synovial fluid (cultural) Nucleic acid detection in synovial fluid (*e.g.*, PCR).

Source: Case definitions of communicable diseases for the ÖGD: Diseases for which, according to the LVO, there is an extended reporting requirement in addition to the IfSG (as of 2009). RKI Epidemiological Bulletin. February 2, 2009 No. 5 (rki.ed).

CSF, cerebrospinal fluid; ELISA, enzyme-linked immunosorbent assay; LB, Lyme borreliosis; NA, not applicable.

Since German recommendations (German Dermatology Society diagnostic guidelines) for LB diagnosis do not require laboratory confirmation for EM, we postulated that clinically diagnosed-only cases were EM, while cases that also had laboratory confirmation were LA or LNB (Hofmann et al. 2017). This conforms with the EUCALB/ESGBOR case definitions and EFNS guidelines for LNB, which recommend laboratory confirmation for all LB manifestations except for EM (Mygland et al. 2010, Rizzoli et al. 2011, Stanek et al. 2011).

### Data sources and variables

To investigate geographic distribution, demographic characteristics, and seasonality of LB, we extracted data for the 5-year period (January 1, 2016 to December 31, 2020), from the online platform SurvStat@RKI 2.0 maintained by the RKI (Robert Koch Institute 2021b). LB cases and incidence were extracted by year, geographic area, notification week, sex, and age for two case definition categories, that is, (1) clinical criteria met (clinically diagnosed LB cases) or (2) clinical and laboratory criteria met (clinically diagnosed and laboratory-confirmed LB cases). Data were extracted for three geographic area levels: nine states corresponding to the Nomenclature of Territorial Units for Statistics 1 (NUTS1); 19 regions, NUTS2; 158 counties and 65 cities (“Landkreis” and “Stadtkreis”), NUTS3. Age was classified into 5-year intervals, but in 1-year intervals for 0–10-year-old children.

### Statistical methods

Data for reported LB cases and incidence were extracted from SurvStat@RKI 2.0 on January 25, 2021. Denominators (population size under consideration) were re-estimated using number of cases and available incidence estimates as state-specific denominators were not made available from RKI. The 95% confidence intervals (CIs) for incidence were calculated using the exact binomial method (Wilson 1927). Average number of cases and average incidence were calculated over the 5-year period (2016−2020) as weighted average of the annual number of cases and weighted average of yearly incidence estimates, respectively, with weights equal to yearly denominators. The 95% CI for average incidence was calculated using a combination of stratum-specific CIs based on the F-distribution (Waller et al. 1994). We chose to calculate CIs following the recommendations by Redelings, where calculation of interval estimates is recommended even when reporting studies of complete populations (Redelings 2012).

Complete case analysis was performed (*i.e.*, only observations with available information for the number of cases and incidence were accounted for). When necessary, we imputed missing denominators as the average of available data (yearly data). We performed analyses using the statistical software R, version 4.0.4 (R Core Team 2016).

## Results

### Time trend and geographic distribution

**FIG. 1.**
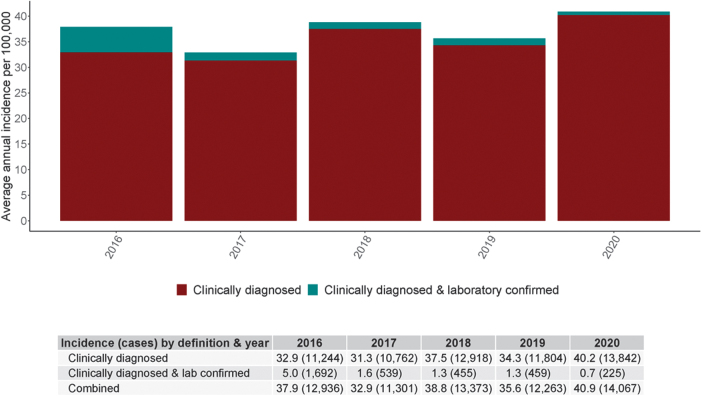
Incidence (per 100,000 person-years, ±95% CI) and cases of clinically diagnosed LB, of clinically diagnosed and laboratory-confirmed LB, and combined (overall) LB reported in nine German states, 2016 − 2020. CI, confidence interval; LB, Lyme borreliosis.

Nine German states (NUTS1) reported LB cases, which were stratified further into 19 regions (NUTS2), and 158 counties and 65 cities (NUTS3). We identified 63,940 LB cases (annual mean: 12,789 cases) reported between 2016–2020. Mean annual incidence during 2016–2020 was 37.2 (95% CI: 36.6–37.9)/100,000 person-years, varying by year from 32.9 (95% CI: 32.3–33.5) in 2017 to 40.9 (95% CI: 40.2–41.6)/100,000 person-years in 2020 (Fig. 1).

Of all the LB cases, 94.7% (60,570/63,940) were clinically diagnosed (range by states: 81−99%) and 5.3% (3370/63,940) were laboratory confirmed (range by states: 1−19%) (Fig. 1). The percentage of cases with laboratory confirmation decreased over time. The state of Berlin reported a higher percentage of laboratory-confirmed cases (19%) than other states (range: 1–7%).

**FIG. 2.**
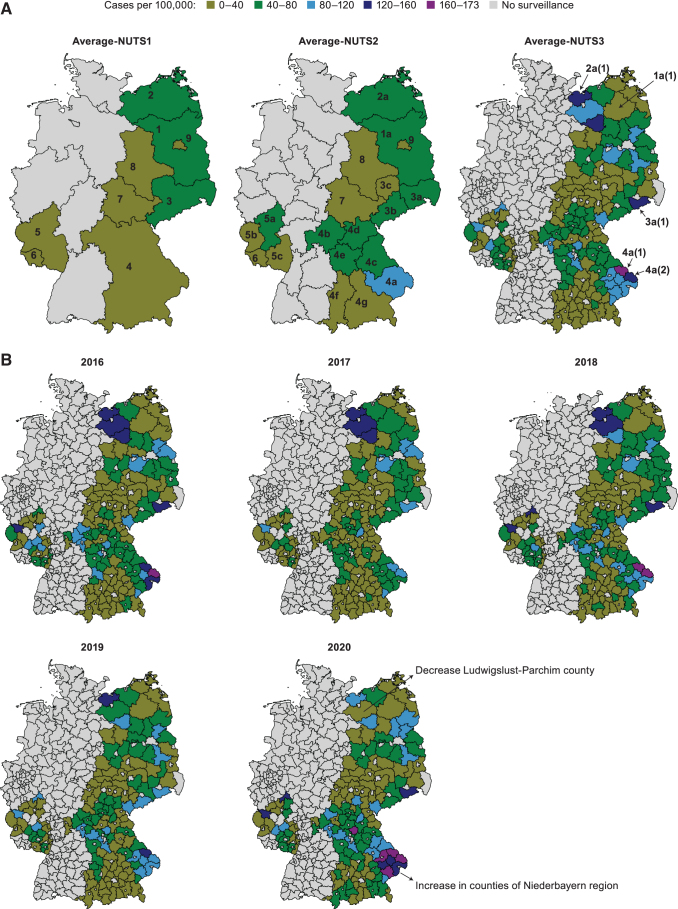
**(A)** Average annual incidence (per 100,000 person-years) of overall LB reported in 9 German states NUTS1, 19 regions NUTS2, and 158 counties NUTS3 over the years 2016−2020. Average-NUTS1: 1 Brandenburg, 2 Mecklenburg-Vorpommern, 3 Saxony, 4 Bavaria, 5 Rhineland-Palatinate, 6 Saarland, 7 Thuringia, 8 Saxony-Anhalt, 9 Berlin. Average-NUTS2: 1a Brandenburg, 2a Mecklenburg-Vorpommern, 3a Dresden, 3b Chemnitz, 3c Leipzig, 4a Niederbayern, 4b Unterfranken, 4c Oberpfalz, 4d Oberfranken, 4e Mittelfranken, 4f Schwaben, 4g Oberbayern, 5a Koblenz, 5b Trier, 5c Rheinhessen-Pfalz, 6 Saarland, 7 Thuringia, 8 Saxony-Anhalt, 9 Berlin. Average-NUTS3: 1a(1) Prignitz County, 2a(1) Nordwestmecklenburg County, 3a(1) Sächsische Schweiz-Osterzgebirge County, 4a(1) Regen County, 4a(2) Freyung-Grafenau County. NUTS, Nomenclature of Territorial Units for Statistics. **(B)** Annual incidence (per 100,000 person-years) of overall LB notified in 158 counties NUTS3, 2016−2020.

Average annual incidence in the nine states varied 2.8-fold between 22.9/100,000 person-years in Berlin and 64.6/100,000 person-years in Brandenburg; in 19 regions, 5.1-fold between 16.8/100,000 person-years in Leipzig and 85.6/100,000 person-years in Niederbayern; and in 158 counties, 59.6-fold between 2.9/100,000 person-years in Burgenlandkreis and 172.8/100,000 person-years in Regen (Table 2 and Fig. 2A). Incidence rates over time were stable at all levels of geographic stratification except for a slight increase in Bavaria in 2020 and a decrease in Mecklenburg-Vorpommern in the last 3 years (Fig. 2B; Supplementary Tables S1–S4).

**Table 2.** Average Annual Incidence (per 100,000 Person-Years, ±95% CI) and Average Annual Cases of Overall Lyme Borreliosis Reported by Nine German State Territorial Units NUTS1, 19 German Region Territorial Units NUTS2, and Five of the 158 German Counties Territorial Units NUTS3 with the Highest Average Annual Incidence Over the Years 2016−2020

	State/NUTS1	Region/NUTS2 (County/NUTS3)	Population	Cases (*n*)	Incidence [95% CI]
1	Brandenburg	Brandenburg	2,510,957	1623	64.6 [61.5–67.9]
		Prignitz County	76,785	97	125.8 [102.1–153.4]
2	Mecklenburg-Vorpommern	Mecklenburg-Vorpommern	1,609,527	873	54.2 [50.7–57.9]
		Nordwestmecklenburg County	157,038	195	124.3 [107.5–143.0]
3	Saxony	—	4,077,048	2038	49.9 [47.8–52.2]
		Chemnitz	1,437,737	886	61.7 [57.7–65.8]
		Dresden	1,598,073	976	61.1 [57.3–65.1]
		Sächsische Schweiz-Osterzgebirge County	245,656	305	124.0 [110.5–138.7]
		Leipzig	1,041,377	175	16.8 [14.4–19.5]
4	Bavaria	—	13,051,461	4728	36.2 [35.2–37.2]
		Niederbayern	1,235,336	1059	85.6 [80.6–90.9]
		Regen County	77,431	134	172.8 [145.1–204.3]
		Freyung-Grafenau County	78,320	118	150.2 [124.5–179.6]
		Unterfranken	1,314,939	596	45.3 [41.8–49.1]
		Oberpfalz	1,107,256	486	43.9 [40.1–47.9]
		Oberfranken	1,065,467	461	43.3 [39.5–47.4]
		Mittelfranken	1,766,146	758	42.9 [39.9–46.0]
		Schwaben	1,883,705	426	22.6 [20.5–24.9]
		Oberbayern	4,678,120	942	20.1 [18.9–21.4]
5	Rhineland-Palatinate	—	4,082,403	1366	33.5 [31.7–35.3]
		Koblenz	1,495,462	689	46.1 [42.7–49.6]
		Trier	531,103	177	33.4 [28.7–38.7]
		Rheinhessen-Pfalz	2,055,777	499	24.3 [22.2–26.5]
6	Saarland	Saarland	991,069	277	28.0 [24.8–31.5]
7	Thuringia	Thuringia	2,143,920	520	24.3 [22.2–26.4]
8	Saxony-Anhalt	Saxony-Anhalt	2,211,429	523	23.6 [21.7–25.8]
9	Berlin	Berlin	3,675,535	842	22.9 [21.4–24.5]
	Total nine states		34,353,347	12,789	37.2 [36.6–37.9]

For Brandenburg, Mecklenburg-Vorpommern, Saarland, Saxony-Anhalt, and Berlin, the area defined as territorial unit region/NUTS2 equals/covers the whole state/NUTS1 area.

CI, confidence interval.

### Demographic characteristics and seasonality

**FIG. 3.**
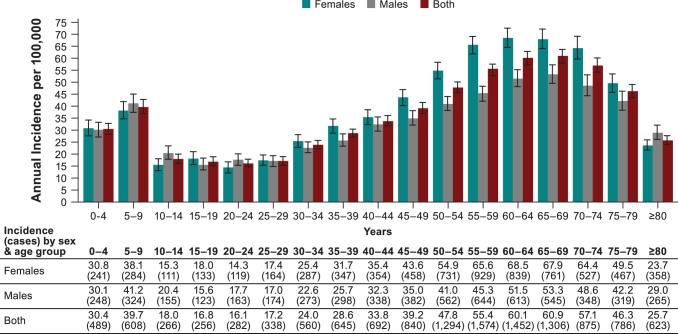
Average annual incidence (per 100,000 person-years, ±95% CI) and average annual cases of overall LB reported in nine German states by age group and sex over the years 2019−2020.

Of the 63,940 LB cases reported during 2016–2020, information on sex was available for 63,134 cases (98.7%) (Supplementary Tables S5 and S6). Females represented 55.3% (34,696/63,134) of all the LB cases. Average annual incidence was 39.9/100,000 person-years (95% CI: 39.1–40.7) in females compared with 33.5/100,000 person-years (95% CI: 32.8–34.2) in males. Incidences of all LB cases and clinically diagnosed cases were higher among females (Supplementary Table S5). Laboratory-confirmed LB cases were more common in males with stable patterns during the study period (Supplementary Table S6). We observed a bimodal age distribution in 2019–2020, with incidence peaks among 5–9-year-old children (higher in males) and 55–74-year-old adults (higher in females) (Fig. 3).

**FIG. 4.**
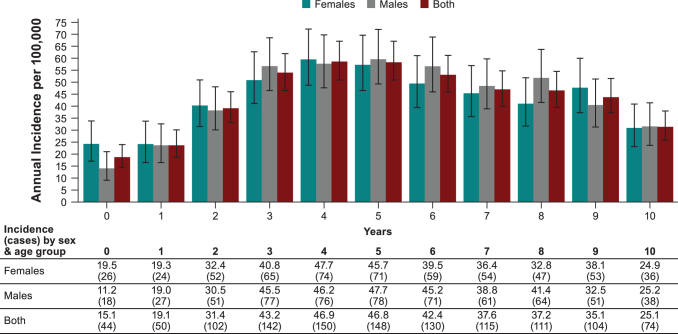
Average annual incidence (per 100,000 person-years, ±95% CI) and average annual cases of overall LB reported in all nine German states in children 0−10 years of age by sex over the years 2019−2020.

Incidence among ≤1-year-old children was approximately the same as among 10–29-year-old individuals (Figs. 3 and 4). Peak incidence among 0–10-year-old children occurred from 3–6 years of age.

**FIG. 5.**
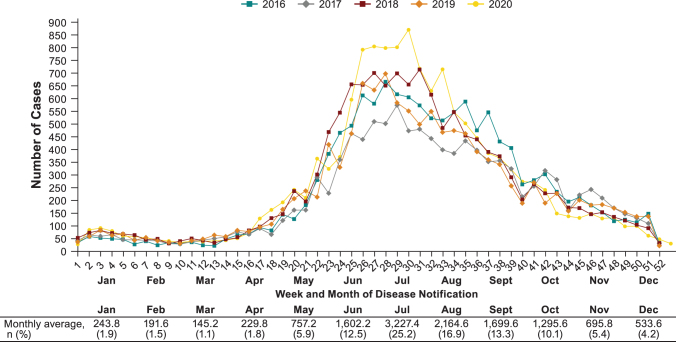
Seasonality of overall LB cases reported in all nine German states by week, month, and year, 2016−2020.

Week of notification of an LB case was determined by the date on which local health departments were informed of the case (Robert Koch Institute 2021a). LB cases were reported throughout the year, with an overall mean of 246 LB weekly cases. During the years 2016–2020, 68% of all LB cases were reported between June and September, peaking at the end of June and beginning of July (week 26–30) with an average of 645 LB weekly cases. Year-to-year variation in seasonality was minor (Fig. 5).

## Discussion

Using surveillance data from the RKI online platform, we examined trends in incidence and geographic distribution of LB in nine federal German states during 2016–2020. LB incidence per geographic unit was highly heterogeneous, with sixfold variation by county. Annual fluctuations in the overall LB cases among the nine federal states were observed; however, three regions (Brandenburg, Mecklenburg-Vorpommern, and Sachsen) reported higher incidences than the average annual incidence of the country consistently during the study period. Out of the nine federal states, Berlin, Brandenburg, Mecklenburg-Vorpommern, Sachsen, and Sachsen-Anhalt have extended notification systems for physicians to also include laboratories, which explains higher reported laboratory-confirmed cases in some federal states (Robert Koch Institute 2017, Enkelmann et al. 2018). The overall incidence remained the same with no clear increase or decrease during the study period, consistent with previous analyses (Enkelmann et al. 2018, Akmatov et al. 2021).

However, we observed highest clinically confirmed LB cases in the first year of the COVID-19 pandemic, 2020. This observation could be attributed to several factors such as increased time spent outdoors and restricted international travel leading to local tourism in high tick density areas in Germany. Contrarily, the pandemic contributed to underreporting of disseminated LB cases. In 2020 in the United States, lower LNB and LA cases were observed versus previous years, which may have been a reflection of underreporting or underdiagnosis (McCormick et al. 2021, Wormser et al. 2021). It is not feasible to ascertain the true impact of the COVID-19 pandemic.

A companion article to this special issue reports the incidence of LB for several countries through public health surveillance (Burn et al. 2023). Directly to the east of Germany, both Poland and Czech Republic had an incidence similar to Germany (40.1/100,000 population per year [PPY] and 54.6/100,000 PPY) from the most recently reported surveillance data. However, the incidence of LB in Germany was substantially lower than other countries within Europe, which included the following: Estonia, Lithuania, Slovenia, and Switzerland (>100 cases/100,000 PPY). Nevertheless, incidences were lower than Germany in Belgium, Bulgaria, Croatia, England, Hungary, Ireland, Norway, Portugal, Romania, Russia, Scotland, and Serbia (<20/100,000 PPY). Most countries displayed similar spatial heterogeneity as Germany. To be sure, comparing incidence rates between countries can be challenging given the differences in reporting systems, access to health care, and distribution of infected ticks.

LB incidence was particularly high in the regions (NUTS2) of West-Mecklenburg and north Brandenburg (Prignitz), Dresden in Saxony, forested areas in Rheinland-Palatinate and Oberbayern, and forested areas in the Niederbayern region of Bavaria. Our data corroborate previous analyses describing German regions with high incidence (Enkelmann et al. 2018, Akmatov et al. 2021, Böhmer et al. 2021), and indicate conducive climatic and environmental conditions in these regions for competent tick-to-host transmission and subsequent transmission to humans (Finch et al. 2014, Seukep et al. 2015, Sharareh et al. 2019).

Despite relatively high incidences identified in our study, underreporting likely occurred, although the extent is unknown. Underreporting of disseminated LB manifestations NB and LA could be explained by complex case definitions based on clinical manifestation and requiring laboratory confirmation (Enkelmann et al. 2018, Böhmer et al. 2021). A 12-month prospective study from 1996–1997 in southern Germany's Würzburg region reported an overall LB annual incidence of 111/100,000 person-years (313 cases), threefold higher than our study (Huppertz et al. 1999). Hospitalization data based on International Classification of Diseases, Tenth Revision (ICD-10) discharge diagnoses are available from the Gesundheitsberichterstattung des Bundes (GBE-BUND; Federal Association for Statutory Health Insurance Physicians [KBV] [2021]) database.

For the years 2010–2018, an average incidence of 14.5 LB hospitalizations per 100,000 was reported, with LB code (ICD-10 codes A69.2, G01 and/or M01.2) as the principal diagnosis for 54% of cases (The Federal Health Reporting Information System 2021). If we assume that 4.4% of cases are hospitalized (range, 3.9 − 5.3%) (Enkelmann et al. 2018) and apply this to a hospitalization incidence of 14.5/100,000, we estimate the combined inpatient and outpatient LB incidence as 330 (range, 273 − 371) per 100,000. This results in an ∼10-fold higher LB incidence compared with the incidence obtained in our study. In addition, three health claim studies (using ICD-10 coding) estimated incidences comparable with GBE-BUND data (Muller et al. 2012, Lohr et al. 2015, Akmatov et al. 2021). Analyses of LB incidence from administrative databases have limitations, including potential for lack of specificity for LB. Nevertheless, the 10-fold incidence difference suggests some degree of underreporting from established surveillance.

Our article highlights the spatial distribution of LB in Germany. Some counties had a higher reported number of cases than entire states from other regions. For instance, Sächsische Schweiz-Osterzgebirge County in Saxony, presumably because of forested and rural areas of high tick density, had more cases than all of Saarland (Brugger et al. 2016), illustrating the importance of small-area microenvironments, human behavior, and sociodemographic factors on eventual transmission to humans.

Age-specific LB incidence was analyzed only for 2019−2020 where we observed bimodal age distribution with a high incidence peak among 5–9-year-old children and 55–74-year-old adults. A similar age distribution has been previously reported (Lohr et al. 2015, Enkelmann et al. 2018). Differences observed by age could be related to factors such as increased outdoor activity levels, which would increase exposure to tick bites, and immunological or biological differences (Skufca and Arima 2012, Enkelmann et al. 2018). In age-stratified incidence data for 0–10-year-old children, we observed the highest incidence among 4–5-year olds, whose daily play routine may cause greater tick bite exposure. For example, “forest kindergartens” are popular in Germany, which are kindergartens for 3–6-year-old children, are located in forested areas, and are almost exclusively outdoors during all seasons (Weisshaar et al. 2006).

More LB cases were reported in females (55%). Incidence among males was higher in the age groups 5–14 and 20–24 years, while in those older than 50 years, incidence was higher among females. Previous European and German studies also found that more LB cases occurred in females (Strle et al. 2002, Fulop and Poggensee 2008, Wilking and Stark 2014, Sajanti et al. 2017, Petrulioniene et al. 2020). Another German study reported that 54% of overall clinically diagnosed cases occurred in females, while more males had acute LNB and LA (Enkelmann et al. 2018). Another study reported higher hospitalization incidence in males (Lohr et al. 2015).

In France, more severe LB cases (hospitalizations) were observed among males (57%) (Septfons et al. 2019). It was suggested that these differences may be attributable to different health-seeking behaviors (Wilking and Stark 2014). For example, females may seek care more readily early during illness, while males seek care following disease progression.

Seasonal distribution of LB cases showed that cases peaked every year in July. The majority of cases were reported between June and September, although there were cases reported during the whole year. The pronounced, stable peak during summer is consistent with previous findings in other countries and in Germany (Lohr et al. 2015, Sajanti et al. 2017, Enkelmann et al. 2018, Septfons et al. 2019, Petrulioniene et al. 2020). The trend is consistent with the data from 2013–2017 (Enkelmann et al. 2018). Week assignment was not based on disease onset or diagnosis date, but on date of notification to the local health department. Thus, we could not account for delay from tick bite to onset of symptoms and diagnosis, something that may have a more pronounced effect on disseminated LB.

The German Dermatology Society diagnostic guidelines define that a clinical evaluation is sufficient to diagnose EM, while further laboratory confirmation is required for atypical EM and disseminated forms (LA, LNB) (Hofmann et al. 2017). Among all the reported LB cases, 95% were based on clinical diagnosis (reflecting likely EM) and 5% on both clinical diagnosis and laboratory confirmation, reflecting likely disseminated LB over the study period. Our findings are consistent with previous German studies (Wilking and Stark 2014, Enkelmann et al. 2018). In a recent study from Bavaria, of all LB cases in the period from 2013–2020, 96.7% were reported as EM, while among laboratory-confirmed LB manifestations, 1.7% were NB and 1.8% were LA. Multiple manifestations for LA were indicated, but atypical EM cases were not described (Böhmer et al. 2021).

We included data for nine reporting federal states, as there are no data from the other seven German states where no LB surveillance exists. However, some German regions with no current surveillance might have a significant burden of LB, especially in forested areas of southwest Germany where tick abundance is also high. Nonetheless, additional factors such as proportion of ticks infected with *B. burgdorferi* and other climatic factors could play crucial roles and require further investigation (Brugger et al. 2016, Ehrmann et al. 2018). A recent health claims analysis from Akmatov et al. (2021) confirms this hypothesis, as it identified substantial burden in nonreporting states.

A strength of our study is that we analyzed spatially granular public surveillance data. Spatial analyses of LB incidence emphasize the importance of examining and presenting data at the most spatially granular unit feasible to accurately reflect localized endemicity. Another strength is that we used the most recent data available, thereby providing current LB epidemiology in Germany. Also, we were able to provide detailed age-stratified incidence data for 0–10-year-old children by year of age in Germany.

Our study had limitations. We were not able to register LB cases by different manifestations, such as EM, LA, or LNB, due to the lack of such granularity in case reporting at the national-level database. Consequently, the assumption that laboratory-confirmed cases in the database represent disseminated LB should be considered an approximation. Also, the place of infection was not available in the database; therefore, stratification by geographic area is based on the place of residence or place of diagnosis. This will introduce some bias into the geographic distribution of cases, which would increase with the granularity of the geographic unit. Another limitation is that age-specific LB incidence data were analyzed only for the recent years, 2019–2020.

## Conclusions

Our study contributes to the understanding of LB in Germany. The data are important to support public health decision-making for investment in future preventive interventions, which can include vaccines in development. The results of our study suggest that LB was stable during the years of the study in nine German federal states where the disease was reported. Nevertheless, we observed microendemic regions of consistently high disease incidence over the study period. Administrative databases suggest that the reported incidences may substantially underestimate true burden. Our results emphasize the importance of differential diagnosis, case reporting, and collating spatially granular data including the geographical area and date where the tick bite occurred to correctly identify areas with the highest incidence and assess the potential for targeted interventions.

Thus, we recommend efforts to improve uniform LB reporting in federal states currently under surveillance, to include all other federal states of Germany in the current surveillance system, to have reporting forms distinguishing EM from LA and LNB, and to collect geographically granular data. This should improve the ability of public health officials to develop the most efficient prevention strategies.

## Supplementary Material

Supplemental data

Supplemental data

Supplemental data

Supplemental data

Supplemental data

Supplemental data

## References

[B1] Akmatov MK, Holstiege J, Dammertz L, Kohring C, et al. Nationwide and local figures on the morbidity of Lyme disease in Germany based on panels doctors accounting data, 2010 to 2019. Central Institute for the Kassena Medical Care in Germany (Zi). Supply Atlas Report No. 21/06 [in German]. Berlin; 2021.

[B2] Böhmer MM, Ens K, Böhm S, Heinzinger S, et al. Epidemiological surveillance of Lyme borreliosis in Bavaria, Germany, 2013–2020. Microorganisms 2021; 9:1872. 34576768 10.3390/microorganisms9091872 PMC8467410

[B3] Brugger K, Boehnke D, Petney T, Dobler G, et al. A density map of the tick-borne encephalitis and Lyme borreliosis vector ixodes ricinus (Acari: Ixodidae) for Germany. J Med Entomol 2016; 53:1292–1302. 27498885 10.1093/jme/tjw116

[B4] Burn L, Tran TMP, Pilz A, Vyse A, et al. Incidence of Lyme borreliosis in Europe from National Surveillance Systems (2005–2020). Vector Borne Zoonotic Dis 2023; 23(4). doi: 10.1089/vbz.2022.0071. PMC10122223 37071405

[B5] Cardenas-de la Garza JA, De la Cruz-Valadez E, Ocampo-Candiani J, Welsh O. Clinical spectrum of Lyme disease. Eur J Clin Microbiol Infect Dis 2019; 38:201–208. 30456435 10.1007/s10096-018-3417-1

[B6] Dehnert M, Fingerle V, Klier C, Talaska T, et al. Seropositivity of Lyme borreliosis and associated risk factors: A population-based study in children and adolescents in Germany (KiGGS). PLoS One 2012; 7:e41321. 22905101 10.1371/journal.pone.0041321 PMC3419690

[B7] Ehrmann S, Ruyts SC, Scherer-Lorenzen M, Bauhus J, et al. Habitat properties are key drivers of Borrelia burgdorferi (s.l.) prevalence in Ixodes ricinus populations of deciduous forest fragments. Parasit Vectors 2018; 11:23. 29310722 10.1186/s13071-017-2590-x PMC5759830

[B8] Enkelmann J, Bohmer M, Fingerle V, Siffczyk C, et al. Incidence of notified Lyme borreliosis in Germany, 2013–2017. Sci Rep 2018; 8:14976. 30297731 10.1038/s41598-018-33136-0 PMC6175818

[B9] Federal Association for Statutory Health Insurance Physicians (KBV). More and more practices are using cameras—Number of video consultations has risen to over one million. 2021. Available at: https://www.kbv.de/html/1150_50419.php

[B10] Finch C, Al-Damluji MS, Krause PJ, Niccolai L, et al. Integrated assessment of behavioral and environmental risk factors for Lyme disease infection on Block Island, Rhode Island. PLoS One 2014; 9:e84758. 24416278 10.1371/journal.pone.0084758 PMC3885597

[B11] Fulop B, Poggensee G. Epidemiological situation of Lyme borreliosis in Germany: Surveillance data from six eastern German states, 2002 to 2006. Parasitol Res 2008; 103 Suppl 1:S117–S120. 19030893 10.1007/s00436-008-1060-y

[B12] Hofmann H, Fingerle V, Hunfeld KP, Huppertz HI, et al. Cutaneous Lyme borreliosis: Guideline of the German Dermatology Society. Ger Med Sci 2017;15:Doc14. 28943834 10.3205/000255 PMC5588623

[B13] Huppertz HI, Bohme M, Standaert SM, Karch H, et al. Incidence of Lyme borreliosis in the Wurzburg region of Germany. Eur J Clin Microbiol Infect Dis 1999; 18:697–703. 10584895 10.1007/s100960050381

[B14] Lohr B, Muller I, Mai M, Norris DE, et al. Epidemiology and cost of hospital care for Lyme borreliosis in Germany: Lessons from a health care utilization database analysis. Ticks Tick Borne Dis 2015; 6:56–62. 25448420 10.1016/j.ttbdis.2014.09.004

[B15] McCormick DW, Kugeler KJ, Marx GE, Jayanthi P, et al. Effects of COVID-19 pandemic on reported Lyme disease, United States, 2020. Emerg Infect Dis 2021; 27:2715–2717. 34545801 10.3201/eid2710.210903 PMC8462321

[B16] Muller I, Freitag MH, Poggensee G, Scharnetzky E, et al. Evaluating frequency, diagnostic quality, and cost of Lyme borreliosis testing in Germany: A retrospective model analysis. Clin Dev Immunol 2012; 2012:595427. 22242037 10.1155/2012/595427 PMC3254124

[B17] Mygland A, Ljøstad U, Fingerle V, Rupprecht T, et al. EFNS guidelines on the diagnosis and management of European Lyme neuroborreliosis. Eur J Neurol 2010; 17:8–16.e1–e4. 19930447 10.1111/j.1468-1331.2009.02862.x

[B18] Petrulioniene A, Radzisauskiene D, Ambrozaitis A, Caplinskas S, et al. Epidemiology of Lyme disease in a highly endemic European zone. Medicina (Kaunas) 2020; 56:115. 32151097 10.3390/medicina56030115 PMC7143858

[B19] Rath PM, Ibershoff B, Mohnhaupt A, Albig J, et al. Seroprevalence of Lyme borreliosis in forestry workers from Brandenburg, Germany. Eur J Clin Microbiol Infect Dis 1996; 15:372–377. 8793394 10.1007/BF01690092

[B20] R Core Team. R: A Language and Environment for Statistical Computing. Vienna, Austria: R Foundation for Statistical Computing, 2016. Available at: https://www.R-project.org

[B21] Redelings MR. Why confidence intervals should be used in reporting studies of complete populations. Open Public Health J 2012; 5:52–54.

[B22] Rizzoli A, Hauffe H, Carpi G, Vourc HG, et al. Lyme borreliosis in Europe. Euro Surveill 2011; 16:19906. 21794218

[B23] Robert Koch Institute. Epidemiologisches Bulletin. Case definitions communicable diseases for the ODG: Diseases for which, according to the LVO, an extended reporting obligation in addition to the IFSG (as of 2009) [in German]. 2009. Available at: https://www.rki.de/DE/Content/Infekt/EpidBull/Archiv/2009/Ausgaben/05_09

[B24] Robert Koch Institute. Notifiable diseases and pathogens [in German]. 2017. Available at: https://www.rki.de/DE/Content/Infekt/IfSG/Meldepflichtige_Krankheiten/Meldepflichtige_Krankheiten_Erreger.pdf?__blob=publicationFile

[B25] Robert Koch Institute. SurvStat@RKI 2.0. 2021a. Available at: https://survstat.rki.de

[B26] Robert Koch Institute. Survstat@RKI 2.0, General Information. 2021b. Available at: https://survstat.rki.de/Content/Instruction/Content.aspx#merkm

[B27] Sajanti E, Virtanen M, Helve O, Kuusi M, et al. Lyme borreliosis in Finland, 1995–2014. Emerg Infect Dis 2017; 23:1282–1288. 28726624 10.3201/eid2308.161273 PMC5547811

[B28] Septfons A, Goronflot T, Jaulhac B, Roussel V, et al. Epidemiology of Lyme borreliosis through two surveillance systems: The National Sentinelles GP Network and the National Hospital Discharge Database, France, 2005 to 2016. Euro Surveill 2019; 24:1800134. 30892181 10.2807/1560-7917.ES.2019.24.11.1800134 PMC6425552

[B29] Seukep SE, Kolivras KN, Hong Y, Li J, et al. An examination of the demographic and environmental variables correlated with Lyme disease emergence in Virginia. Ecohealth 2015; 12:634–644. 26163019 10.1007/s10393-015-1034-3

[B30] Sharareh N, Behler RP, Roome AB, Shepherd J, et al. Risk factors of Lyme disease: An intersection of environmental ecology and systems science. Healthcare (Basel) 2019; 7:66. doi: 10.3390/healthcare7020066. 31052225 PMC6627148

[B31] Skufca J, Arima Y. Sex, gender and emerging infectious disease surveillance: A leptospirosis case study. Western Pac Surveill Response J 2012; 3:37–39. 23908921 10.5365/WPSAR.2012.3.3.001 PMC3731007

[B32] Stanek G, Fingerle V, Hunfeld KP, Jaulhac B, et al. Lyme borreliosis: Clinical case definitions for diagnosis and management in Europe. Clin Microbiol Infect 2011; 17:69–79. 20132258 10.1111/j.1469-0691.2010.03175.x

[B33] Strle F, Videcnik J, Zorman P, Cimperman J, et al. Clinical and epidemiological findings for patients with erythema migrans. Comparison of cohorts from the years 1993 and 2000. Wien Klin Wochenschr 2002; 114:493–497. 12422589

[B34] The Federal Health Reporting Information System. Federal Health Reporting. Lyme Borreliosis—Diagnostic data of the hospitals. 2021. Available at: www.gbe-bund.de

[B35] Waller JL, Addy CL, Jackson KL, Garrison CZ. Confidence intervals for weighted proportions. Stat Med 1994; 13:1071–1082. 8073202 10.1002/sim.4780131009

[B36] Weisshaar E, Schaefer A, Scheidt RR, Bruckner T, et al. Epidemiology of tick bites and borreliosis in children attending kindergarten or so-called “forest kindergarten” in southwest Germany. J Invest Dermatol 2006; 126:584–590. 16410779 10.1038/sj.jid.5700160

[B37] Wilking H, Fingerle V, Klier C, Thamm M, et al. Antibodies against Borrelia burgdorferi sensu lato among adults, Germany, 2008–2011. Emerg Infect Dis 2015; 21:107–110. 25531140 10.3201/eid2101.140009 PMC4285254

[B38] Wilking H, Stark K. Trends in surveillance data of human Lyme borreliosis from six federal states in eastern Germany, 2009–2012. Ticks Tick Borne Dis 2014; 5:219–224. 24656810 10.1016/j.ttbdis.2013.10.010

[B39] Wilson EB. Probable inference, the law of succession, and statistical inference. J Am Stat Assoc 1927; 22:209–212.

[B40] Wormser GP, Jacobson E, Shanker EM. Negative impact of the COVID-19 pandemic on the timely diagnosis of tick-borne infections. Diagn Microbiol Infect Dis 2021; 99:115226. 33070027 10.1016/j.diagmicrobio.2020.115226 PMC7518953

[B41] Woudenberg T, Bohm S, Bohmer M, Katz K, et al. Dynamics of Borrelia burgdorferi-specific antibodies: Seroconversion and seroreversion between two population-based, cross-sectional surveys among adults in Germany. Microorganisms 2020; 8:1859. 33255673 10.3390/microorganisms8121859 PMC7761102

