# Supplementary material for: Incidence of Lyme Borreliosis in Germany: Exploring Observed Trends Over Time Using Public Surveillance Data, 2016–2020
Source: Vector Borne Zoonotic Dis. 2023 Apr 12;23(4):237–46. doi: 10.1089/vbz.2022.0046 (PMC10122258; doi:10.1089/vbz.2022.0046)
Supplement: Supplemental data [file Supp_TableS1.pdf]

Table S1. Cases and incidence (per 100,000 person-time, ± 95% CI) of overall Lyme borreliosis notified by year and by 9 German States Territorial Units NUTS1, yearly 2016–2020.

	2016		2017		2018		2019		2020	
**State/ NUTS1**	**Cases (population)**	**Incidence [95% CI]**	**Cases (population)**	**Incidence [95% CI]**	**Cases (population)**	**Incidence [95% CI]**	**Cases (population)**	**Incidence [95% CI]**	**Cases (population)**	**Incidence [95% CI]**
Bavaria	4,592(12,931,569)	35.51 [34.5;36.55]	3,538(12,997,796)	27.22 [26.34;28.13]	5,014(13,077,726)	38.34 [37.29;39.42]	4,255(13,124,614)	32.42 [31.46;33.41]	6,231(13,123,420)	47.48 [46.32;48.67]
Berlin	900 (3,574,265)	25.18 [23.59;26.88]	763 (3,711,089)	20.56 [19.15;22.07]	755 (3,748,759)	20.14 [18.75;21.63]	851 (3,669,685)	23.19 [21.68;24.8]	943 (3,669,261)	25.7 [24.11;27.39]
Brandenburg	1,673 (2,494,781)	67.06 [63.92;70.35]	1,743 (2,503,951)	69.61 [66.42;72.95]	1,556 (2,512,108)	61.94 [58.94;65.09]	1,535 (2,521,768)	60.87 [57.9;63.99]	1,608 (2,521,957)	63.76 [60.72;66.95]
Mecklenburg-Vorpommern	973 (1,610,660)	60.41 [56.73;64.33]	1,087 (1,611,086)	67.47 [63.58;71.6]	852 (1,609,673)	52.93 [49.49;56.6]	802 (1,608,181)	49.87 [46.54;53.44]	649 (1,608,028)	40.36 [37.37;43.59]
Rhineland-Palatinate	1,471 (4,065,782)	36.18 [34.38;38.08]	1,084 (4,073,657)	26.61 [25.07;28.24]	1,580 (4,084,798)	38.68 [36.82;40.63]	1,185 (4,093,264)	28.95 [27.35;30.65]	1,510 (4,094,360)	36.88 [35.07;38.79]
Saarland	197 (996,459)	19.77 [17.2;22.73]	196 (994,419)	19.71 [17.14;22.67]	375 (990,491)	37.86 [34.22;41.89]	288 (986,977)	29.18 [26;32.75]	332 (986,920)	33.64 [30.21;37.46]
Saxony	2,057 (4,082,159)	50.39 [48.26;52.61]	1,867 (4,080,874)	45.75 [43.72;47.87]	2,146 (4,078,297)	52.62 [50.44;54.89]	2,309 (4,072,310)	56.7 [54.43;59.06]	1,809 (4,071,573)	44.43 [42.43;46.52]
Saxony-Anhalt	506 (2,235,970)	22.63 [20.74;24.69]	573 (2,222,653)	25.78 [23.75;27.98]	530 (2,208,333)	24 [22.04;26.13]	503 (2,194,590)	22.92 [21;25.01]	502 (2,195,015)	22.87 [20.96;24.96]
Thuringia	567 (2,158,356)	26.27 [24.2;28.52]	450 (2,151,052)	20.92 [19.07;22.94]	565 (2,143,399)	26.36 [24.27;28.62]	535 (2,133,174)	25.08 [23.04;27.3]	483 (2,133,392)	22.64 [20.71;24.75]
Total	12,936(34,150,001)	37.88 [37.23;38.54]	11,301(34,346,577)	32.9 [32.3;33.51]	13,373(34,453,585)	38.81 [38.16;39.48]	12,263(34,404,564)	35.64 [35.02;36.28]	14,067(34,403,927)	40.89 [40.22;41.57]
