# Supplementary material for: Incidence of Lyme Borreliosis in Germany: Exploring Observed Trends Over Time Using Public Surveillance Data, 2016–2020
Source: Vector Borne Zoonotic Dis. 2023 Apr 12;23(4):237–46. doi: 10.1089/vbz.2022.0046 (PMC10122258; doi:10.1089/vbz.2022.0046)
Supplement: Supplemental data [file Supp_TableS4.pdf]

Table S4. Cases and incidence (per 100,000 person-time, ± 95% CI) of overall Lyme borreliosis notified by year and by German cities NUTS3, yearly 2016–2020.

			2016		2017		2018		2019		2020	
State/ NUTS1	Region/ NUTS2	City	Cases (population)	Incidence [95% CI]	Cases (population)	Incidence [95% CI]	Cases (population)	Incidence [95% CI]	Cases (population)	Incidence [95% CI]	Cases (population)	Incidence [95% CI]
Thuringia	Thuringia	City of Weimar	13 (64,356)	20.2 [11.81;34.56]	7 (64,397)	10.87 [5.27;22.44]	14 (65,086)	21.51 [12.81;36.11]	20 (65,232)	30.66 [19.85;47.36]	19 (65,225)	29.13 [18.65;45.5]
Thuringia	Thuringia	City of Suhl	46 (35,609)	129.18 [96.87;172.25]	30 (35,166)	85.31 [59.77;121.76]	35 (34,836)	100.47 [72.25;139.69]	52 (36,788)	141.35 [107.81;185.3]	48 (36,790)	130.47 [98.43;172.93]
Thuringia	Thuringia	City of Jena	28 (110,323)	25.38 [17.56;36.68]	21 (111,111)	18.9 [12.36;28.89]	28 (111,421)	25.13 [17.39;36.32]	23 (111,326)	20.66 [13.77;31]	17 (111,329)	15.27 [9.53;24.45]
Thuringia	Thuringia	City of Gera	16 (94,731)	16.89 [10.4;27.44]	13 (94,891)	13.7 [8.01;23.44]	13 (94,135)	13.81 [8.07;23.63]	20 (93,110)	21.48 [13.91;33.18]	21 (93,126)	22.55 [14.75;34.47]
Thuringia	Thuringia	City of Erfurt	5 (210,970)	2.37 [1.01;5.55]	6 (212,766)	2.82 [1.29;6.15]	14 (213,740)	6.55 [3.9;11]	15 (213,980)	7.01 [4.25;11.57]	2 (215,054)	0.93 [0.26;3.39]
Thuringia	Thuringia	City of Eisenach	17 (42,585)	39.92 [24.93;63.93]	8 (42,712)	18.73 [9.49;36.96]	13 (42,373)	30.68 [17.93;52.49]	22 (42,251)	52.07 [34.39;78.83]	11 (42,243)	26.04 [14.54;46.63]
Saxony-Anhalt	Saxony-Anhalt	City of Magdeburg	69 (238,095)	28.98 [22.9;36.67]	84 (238,501)	35.22 [28.45;43.6]	60 (238,663)	25.14 [19.53;32.36]	71 (237,538)	29.89 [23.7;37.7]	4 (238,095)	1.68 [0.65;4.32]
Saxony-Anhalt	Saxony-Anhalt	City of Halle	23 (238,095)	9.66 [6.44;14.5]	39 (239,117)	16.31 [11.93;22.29]	35 (239,234)	14.63 [10.52;20.35]	21 (238,636)	8.8 [5.76;13.45]	45 (238,727)	18.85 [14.09;25.22]
Saxony-Anhalt	Saxony-Anhalt	City of Dessau-Roßlau	11 (82,521)	13.33 [7.44;23.87]	9 (82,117)	10.96 [5.77;20.83]	10 (81,235)	12.31 [6.69;22.66]	3 (80,000)	3.75 [1.28;11.03]	10 (80,128)	12.48 [6.78;22.97]
Saxony	Leipzig	City of Leipzig	65 (571,178)	11.38 [8.93;14.5]	51 (582,192)	8.76 [6.66;11.52]	62 (587,678)	10.55 [8.23;13.52]	75 (593,354)	12.64 [10.08;15.84]	40 (593,472)	6.74 [4.95;9.18]
Saxony	Dresden	City of Görlitz	64 (258,377)	24.77 [19.4;31.63]	74 (256,588)	28.84 [22.98;36.2]	158 (254,880)	61.99 [53.05;72.43]	180 (252,738)	71.22 [61.55;82.41]	61 (252,693)	24.14 [18.8;31]
Saxony	Dresden	City of Dresden	310 (547,220)	56.65 [50.69;63.31]	295 (551,093)	53.53 [47.76;59.99]	309 (554,658)	55.71 [49.84;62.28]	290 (556,729)	52.09 [46.43;58.44]	355 (556,775)	63.76 [57.47;70.74]
Saxony	Chemnitz	City of Chemnitz	166 (246,364)	67.38 [57.88;78.43]	120 (246,863)	48.61 [40.66;58.12]	159 (247,240)	64.31 [55.06;75.11]	182 (246,345)	73.88 [63.9;85.42]	73 (246,372)	29.63 [23.57;37.25]
Rhineland-Palatinate	Rheinhessen-Pfalz	City of Zweibrücken	11 (34,429)	31.95 [17.84;57.21]	7 (34,263)	20.43 [9.9;42.17]	18 (34,208)	52.62 [33.29;83.17]	10 (34,188)	29.25 [15.89;53.84]	12 (34,198)	35.09 [20.07;61.33]
Rhineland-Palatinate	Rheinhessen-Pfalz	City of Worms	8 (82,559)	9.69 [4.91;19.12]	1 (83,333)	1.2 [0.21;6.8]	2 (83,333)	2.4 [0.66;8.75]	2 (83,682)	2.39 [0.66;8.71]	2 (83,682)	2.39 [0.66;8.71]
Rhineland-Palatinate	Trier	City of Trier	13 (110,076)	11.81 [6.9;20.21]	4 (109,890)	3.64 [1.42;9.36]	9 (110,701)	8.13 [4.28;15.45]	12 (111,524)	10.76 [6.16;18.81]	10 (111,483)	8.97 [4.87;16.51]
Rhineland-Palatinate	Rheinhessen-Pfalz	City of Speyer	NA (NA)	NA [NA;NA]	1 (51,020)	1.96 [0.35;11.1]	1 (50,505)	1.98 [0.35;11.22]	3 (50,590)	5.93 [2.02;17.44]	3 (50,590)	5.93 [2.02;17.44]
Rhineland-Palatinate	Rheinhessen-Pfalz	City of Pirmasens	7 (40,416)	17.32 [8.39;35.75]	2 (40,650)	4.92 [1.35;17.94]	19 (40,400)	47.03 [30.11;73.45]	10 (40,225)	24.86 [13.5;45.76]	10 (40,225)	24.86 [13.5;45.76]
Rhineland-Palatinate	Rheinhessen-Pfalz	City of Neustadt a.d.Weinstraße	7 (53,191)	13.16 [6.37;27.16]	8 (53,369)	14.99 [7.6;29.58]	14 (53,151)	26.34 [15.69;44.21]	4 (53,262)	7.51 [2.92;19.31]	4 (53,262)	7.51 [2.92;19.31]
Rhineland-Palatinate	Rheinhessen-Pfalz	City of Mainz	17 (213,568)	7.96 [4.97;12.75]	17 (215,190)	7.9 [4.93;12.65]	29 (217,066)	13.36 [9.3;19.19]	25 (218,531)	11.44 [7.75;16.89]	15 (218,659)	6.86 [4.16;11.32]
Rhineland-Palatinate	Rheinhessen-Pfalz	City of Ludwigshafen	2 (166,667)	1.2 [0.33;4.38]	13 (168,394)	7.72 [4.51;13.21]	32 (171,032)	18.71 [13.25;26.41]	15 (172,216)	8.71 [5.28;14.37]	43 (172,276)	24.96 [18.53;33.62]
Rhineland-Palatinate	Rheinhessen-Pfalz	City of Landau i.d.Pfalz	2 (45,977)	4.35 [1.19;15.86]	2 (46,296)	4.32 [1.18;15.75]	3 (46,656)	6.43 [2.19;18.9]	8 (46,893)	17.06 [8.64;33.66]	6 (46,875)	12.8 [5.87;27.93]
Rhineland-Palatinate	Koblenz	City of Koblenz	6 (113,636)	5.28 [2.42;11.52]	1 (113,636)	0.88 [0.16;4.98]	4 (113,960)	3.51 [1.36;9.03]	4 (113,960)	3.51 [1.36;9.03]	6 (114,068)	5.26 [2.41;11.48]
Rhineland-Palatinate	Rheinhessen-Pfalz	City of Kaiserslautern	29 (99,315)	29.2 [20.33;41.93]	31 (99,678)	31.1 [21.91;44.14]	54 (99,852)	54.08 [41.45;70.55]	42 (100,024)	41.99 [31.07;56.75]	50 (100,020)	49.99 [37.92;65.89]

Rhineland-Palatinate	Rheinhessen-Pfalz	City of Frankenthal	NA (NA)	NA [NA;NA]	6 (48,426)	12.39 [5.68;27.03]	4 (48,544)	8.24 [3.2;21.19]	5 (48,780)	10.25 [4.38;23.99]	7 (48,747)	14.36 [6.96;29.64]
Mecklenburg-Vorpommern	Mecklenburg-Vorpommern	City of Schwerin	139 (95,671)	145.29 [123.07;171.51]	136 (95,795)	141.97 [120.04;167.9]	85 (95,818)	88.71 [71.76;109.67]	75 (95,651)	78.41 [62.56;98.27]	26 (95,659)	27.18 [18.55;39.82]
Mecklenburg-Vorpommern	Mecklenburg-Vorpommern	City of Rostock	25 (207,469)	12.05 [8.16;17.79]	18 (208,333)	8.64 [5.47;13.66]	9 (208,817)	4.31 [2.27;8.19]	60 (209,205)	28.68 [22.28;36.91]	23 (209,281)	10.99 [7.32;16.49]
Brandenburg	Brandenburg	City of Potsdam	96 (171,797)	55.88 [45.77;68.23]	113 (175,711)	64.31 [53.5;77.31]	86 (178,091)	48.29 [39.11;59.63]	59 (180,318)	32.72 [25.37;42.2]	84 (180,335)	46.58 [37.63;57.66]
Brandenburg	Brandenburg	City of Frankfurt (Oder)	37 (58,194)	63.58 [46.13;87.62]	29 (58,233)	49.8 [34.68;71.51]	37 (57,876)	63.93 [46.39;88.1]	34 (57,754)	58.87 [42.13;82.25]	23 (57,745)	39.83 [26.54;59.76]
Brandenburg	Brandenburg	City of Cottbus	29 (100,416)	28.88 [20.11;41.47]	11 (101,010)	10.89 [6.08;19.5]	19 (100,211)	18.96 [12.14;29.61]	13 (99,693)	13.04 [7.62;22.31]	22 (99,683)	22.07 [14.58;33.42]
Brandenburg	Brandenburg	City of Brandenburg a.d.Havel	82 (71,666)	114.42 [92.2;141.99]	101 (71,886)	140.5 [115.65;170.67]	95 (72,123)	131.72 [107.77;160.98]	82 (72,183)	113.6 [91.54;140.97]	110 (72,183)	152.39 [126.46;183.62]
Berlin	Berlin	City of Berlin Treptow-Köpenick	57 (250,770)	22.73 [17.55;29.45]	74 (265,043)	27.92 [22.24;35.05]	82 (269,737)	30.4 [24.49;37.73]	69 (266,512)	25.89 [20.46;32.76]	80 (266,489)	30.02 [24.12;37.36]
Berlin	Berlin	City of Berlin Tempelhof-Schöneberg	54 (336,868)	16.03 [12.29;20.91]	69 (348,661)	19.79 [15.64;25.04]	62 (351,474)	17.64 [13.76;22.61]	54 (341,772)	15.8 [12.11;20.61]	77 (341,767)	22.53 [18.03;28.15]
Berlin	Berlin	City of Berlin Steglitz-Zehlendorf	99 (293,943)	33.68 [27.67;41]	73 (307,110)	23.77 [18.91;29.88]	84 (308,031)	27.27 [22.03;33.76]	69 (302,234)	22.83 [18.04;28.89]	77 (302,198)	25.48 [20.39;31.84]
Berlin	Berlin	City of Berlin Spandau	32 (232,727)	13.75 [9.74;19.41]	34 (242,165)	14.04 [10.05;19.62]	32 (243,161)	13.16 [9.32;18.58]	32 (238,984)	13.39 [9.49;18.9]	33 (238,957)	13.81 [9.83;19.39]
Berlin	Berlin	City of Berlin Reinickendorf	50 (253,550)	19.72 [14.96;25.99]	36 (263,543)	13.66 [9.87;18.91]	23 (264,977)	8.68 [5.78;13.03]	54 (259,740)	20.79 [15.94;27.12]	43 (259,662)	16.56 [12.3;22.3]
Berlin	Berlin	City of Berlin Pankow	170 (386,364)	44 [37.87;51.13]	153 (402,314)	38.03 [32.46;44.55]	111 (407,041)	27.27 [22.65;32.84]	155 (398,048)	38.94 [33.27;45.57]	168 (398,010)	42.21 [36.29;49.09]
Berlin	Berlin	City of Berlin Neukölln	61 (322,410)	18.92 [14.73;24.3]	61 (329,374)	18.52 [14.42;23.79]	51 (330,739)	15.42 [11.73;20.27]	51 (321,159)	15.88 [12.08;20.88]	58 (321,152)	18.06 [13.97;23.34]
Berlin	Berlin	City of Berlin Mitte	56 (363,636)	15.4 [11.86;20]	32 (377,804)	8.47 [6;11.96]	39 (383,481)	10.17 [7.44;13.9]	57 (375,247)	15.19 [11.73;19.68]	56 (375,335)	14.92 [11.49;19.37]
Berlin	Berlin	City of Berlin Marzahn-Hellersdorf	124 (256,145)	48.41 [40.61;57.71]	90 (266,667)	33.75 [27.46;41.48]	97 (268,773)	36.09 [29.59;44.02]	103 (262,956)	39.17 [32.3;47.5]	127 (262,940)	48.3 [40.6;57.46]
Berlin	Berlin	City of Berlin Lichtenberg	53 (274,754)	19.29 [14.75;25.23]	42 (286,299)	14.67 [10.85;19.83]	63 (290,456)	21.69 [16.96;27.75]	88 (286,552)	30.71 [24.93;37.83]	72 (286,510)	25.13 [19.96;31.64]
Berlin	Berlin	City of Berlin Friedrichshain-Kreuzberg	103 (277,853)	37.07 [30.57;44.95]	61 (283,985)	21.48 [16.72;27.59]	74 (289,175)	25.59 [20.39;32.12]	85 (282,017)	30.14 [24.38;37.26]	125 (282,040)	44.32 [37.2;52.8]
Berlin	Berlin	City of Berlin Charlottenburg-Wilmersdorf	41 (325,914)	12.58 [9.27;17.06]	38 (338,681)	11.22 [8.18;15.4]	37 (341,328)	10.84 [7.86;14.94]	34 (334,317)	10.17 [7.28;14.21]	27 (334,572)	8.07 [5.55;11.74]
Bavaria	Unterfranken	City of Würzburg	82 (126,018)	65.07 [52.43;80.75]	16 (126,683)	12.63 [7.77;20.52]	9 (127,841)	7.04 [3.7;13.38]	9 (128,023)	7.03 [3.7;13.36]	51 (127,948)	39.86 [30.32;52.4]
Bavaria	Oberpfalz	City of Weiden i.d.OPf.	12 (42,493)	28.24 [16.16;49.36]	10 (42,535)	23.51 [12.77;43.28]	16 (42,519)	37.63 [23.16;61.12]	16 (42,746)	37.43 [23.04;60.8]	11 (42,735)	25.74 [14.37;46.09]
Bavaria	Niederbayern	City of Straubing	4 (47,114)	8.49 [3.3;21.83]	3 (47,619)	6.3 [2.14;18.52]	7 (47,782)	14.65 [7.1;30.24]	16 (47,790)	33.48 [20.61;54.38]	21 (47,792)	43.94 [28.74;67.17]
Bavaria	Unterfranken	City of Schweinfurt	13 (52,717)	24.66 [14.41;42.19]	3 (53,476)	5.61 [1.91;16.49]	2 (54,054)	3.7 [1.01;13.49]	4 (53,405)	7.49 [2.91;19.26]	3 (53,381)	5.62 [1.91;16.52]
Bavaria	Mittelfranken	City of Schwabach	NA (NA)	NA [NA;NA]	NA (NA)	NA [NA;NA]	NA (NA)	NA [NA;NA]	NA (NA)	NA [NA;NA]	4 (40,984)	9.76 [3.8;25.09]
Bavaria	Oberbayern	City of Rosenheim	3 (62,630)	4.79 [1.63;14.08]	3 (63,025)	4.76 [1.62;14]	2 (63,291)	3.16 [0.87;11.52]	NA (NA)	NA [NA;NA]	7 (63,579)	11.01 [5.33;22.73]
Bavaria	Oberpfalz	City of Regensburg	41 (148,658)	27.58 [20.33;37.41]	21 (150,862)	13.92 [9.11;21.28]	17 (152,603)	11.14 [6.96;17.84]	5 (152,905)	3.27 [1.4;7.66]	31 (153,086)	20.25 [14.27;28.74]
Bavaria	Niederbayern	City of Passau	NA (NA)	NA [NA;NA]	41 (51,781)	79.18 [58.38;107.39]	51 (52,469)	97.2 [73.94;127.77]	40 (52,805)	75.75 [55.64;103.13]	87 (52,804)	164.76 [133.6;203.17]
Bavaria	Mittelfranken	City of Nürnberg	113 (511,544)	22.09 [18.38;26.56]	91 (515,289)	17.66 [14.39;21.68]	127 (518,367)	24.5 [20.59;29.15]	113 (518,349)	21.8 [18.13;26.21]	140 (518,327)	27.01 [22.89;31.87]
Bavaria	Oberbayern	City of München	55 (1,462,766)	3.76 [2.89;4.89]	65 (1,457,399)	4.46 [3.5;5.68]	115 (1,470,588)	7.82 [6.52;9.39]	79 (1,484,962)	5.32 [4.27;6.63]	154 (1,483,622)	10.38 [8.87;12.15]

Bavaria	Schwaben	City of Memmingen	6 (43,290)	13.86 [6.35;30.24]	2 (43,478)	4.6 [1.26;16.77]	10 (43,840)	22.81 [12.39;41.99]	5 (44,092)	11.34 [4.84;26.55]	6 (44,085)	13.61 [6.24;29.69]
Bavaria	Niederbayern	City of Landshut	26 (70,024)	37.13 [25.34;54.4]	23 (71,185)	32.31 [21.53;48.48]	32 (72,398)	44.2 [31.31;62.39]	31 (73,408)	42.23 [29.75;59.93]	48 (73,406)	65.39 [49.33;86.68]
Bavaria	Oberbayern	City of Ingolstadt	14 (133,588)	10.48 [6.24;17.59]	24 (135,211)	17.75 [11.93;26.41]	36 (136,986)	26.28 [18.98;36.38]	29 (137,376)	21.11 [14.7;30.32]	37 (137,393)	26.93 [19.54;37.11]
Bavaria	Oberfranken	City of Hof	NA (NA)	NA [NA;NA]	2 (45,977)	4.35 [1.19;15.86]	4 (45,924)	8.71 [3.39;22.4]	1 (45,872)	2.18 [0.38;12.35]	6 (45,837)	13.09 [6;28.56]
Bavaria	Mittelfranken	City of Fürth	2 (125,786)	1.59 [0.44;5.8]	1 (126,582)	0.79 [0.14;4.48]	1 (128,205)	0.78 [0.14;4.42]	9 (128,571)	7 [3.68;13.3]	7 (128,440)	5.45 [2.64;11.25]
Bavaria	Mittelfranken	City of Erlangen	41 (110,245)	37.19 [27.42;50.44]	25 (111,012)	22.52 [15.26;33.24]	59 (111,954)	52.7 [40.86;67.97]	28 (112,540)	24.88 [17.22;35.96]	37 (112,530)	32.88 [23.86;45.31]
Bavaria	Oberfranken	City of Coburg	6 (41,068)	14.61 [6.7;31.87]	9 (41,228)	21.83 [11.49;41.49]	2 (41,237)	4.85 [1.33;17.68]	15 (41,073)	36.52 [22.13;60.25]	4 (41,068)	9.74 [3.79;25.04]
Bavaria	Oberfranken	City of Bayreuth	NA (NA)	NA [NA;NA]	2 (74,074)	2.7 [0.74;9.84]	3 (74,627)	4.02 [1.37;11.82]	10 (74,794)	13.37 [7.26;24.61]	15 (74,776)	20.06 [12.16;33.1]
Bavaria	Oberfranken	City of Bamberg	45 (75,745)	59.41 [44.41;79.48]	41 (77,184)	53.12 [39.16;72.05]	49 (77,593)	63.15 [47.77;83.47]	14 (77,391)	18.09 [10.78;30.37]	14 (77,391)	18.09 [10.78;30.37]
Bavaria	Schwaben	City of Augsburg	15 (289,575)	5.18 [3.14;8.55]	18 (292,683)	6.15 [3.89;9.72]	11 (294,906)	3.73 [2.08;6.68]	6 (297,030)	2.02 [0.93;4.41]	20 (296,736)	6.74 [4.36;10.41]
Bavaria	Mittelfranken	City of Ansbach	15 (41,528)	36.12 [21.89;59.59]	17 (41,656)	40.81 [25.48;65.35]	8 (41,841)	19.12 [9.69;37.73]	14 (41,804)	33.49 [19.95;56.21]	6 (41,812)	14.35 [6.58;31.31]
Bavaria	Oberpfalz	City of Amberg	4 (42,328)	9.45 [3.67;24.3]	1 (42,194)	2.37 [0.42;13.42]	1 (42,017)	2.38 [0.42;13.48]	1 (42,194)	2.37 [0.42;13.42]	5 (42,194)	11.85 [5.06;27.74]
