# Supplementary material for: Incidence of Lyme Borreliosis in Germany: Exploring Observed Trends Over Time Using Public Surveillance Data, 2016–2020
Source: Vector Borne Zoonotic Dis. 2023 Apr 12;23(4):237–46. doi: 10.1089/vbz.2022.0046 (PMC10122258; doi:10.1089/vbz.2022.0046)
Supplement: Supplemental data [file Supp_TableS5.pdf]

Table S5. Average annual cases and average annual incidence (per 100,000 person-years, ± 95% CI) of overall Lyme borreliosis notified by 19 German Regions Territorial Units NUTS2 and by sex, over years 2016–2020.

	Females		Males	
**Region/ NUTS2**	**Cases (population)**	**Incidence [95% CI]**	**Cases (population)**	**Incidence [95% CI]**
Territorial unit Mittelfranken	417 (895,706)	46.6 [42.93; 50.52]	337 (870,479)	38.67 [35.28; 42.31]
Territorial unit Niederbayern	554 (616,506)	89.8 [83.69; 96.27]	481 (618,811)	77.64 [71.95; 83.68]
Territorial unit Oberbayern	491 (2,364,004)	20.76 [19.25; 22.36]	430 (2,314,379)	18.56 [17.13; 20.09]
Territorial unit Oberfranken	252 (541,101)	46.57 [41.88; 51.67]	208 (524,354)	39.63 [35.24; 44.44]
Territorial unit Oberpfalz	251 (555,721)	45.2 [40.66; 50.14]	227 (551,574)	41.16 [36.82; 45.89]
Territorial unit Schwaben	223 (946,480)	23.54 [21.01; 26.3]	193 (937,155)	20.55 [18.19; 23.15]
Territorial unit Unterfranken	299 (663,703)	45.02 [40.84; 49.52]	256 (651,271)	39.37 [35.44; 43.64]
Territorial unit Berlin	474 (1,864,028)	25.47 [23.58; 27.47]	366 (1,811,746)	20.23 [18.53; 22.05]
Territorial unit Brandenburg	907 (1,271,915)	71.33 [67.48; 75.34]	714 (1,238,994)	57.68 [54.18; 61.35]
Territorial unit Mecklenburg-Vorpommern	478 (815,689)	58.65 [54.33; 63.24]	392 (793,879)	49.35 [45.34; 53.63]
Territorial unit Koblenz	351 (757,230)	46.3 [42.32; 50.57]	327 (738,297)	44.32 [40.38; 48.55]
Territorial unit Rheinhessen-Pfalz	266 (1,042,314)	25.56 [23.05; 28.28]	222 (1,013,621)	21.86 [19.51; 24.43]
Territorial unit Trier	88 (266,773)	33.14 [27.61; 39.5]	87 (264,340)	32.76 [27.22; 39.15]
Territorial unit Saarland	144 (504,561)	28.66 [24.9; 32.86]	112 (486,432)	23.15 [19.73; 27.02]
Territorial unit Chemnitz	506 (731,657)	69.14 [64.17; 74.39]	381 (706,085)	53.91 [49.46; 58.66]
Territorial unit Dresden	567 (807,679)	70.18 [65.4; 75.22]	409 (790,334)	51.8 [47.67; 56.21]
Territorial unit Leipzig	96 (529,099)	18.15 [15.21; 21.5]	79 (512,305)	15.38 [12.66; 18.54]
Territorial unit Saxony-Anhalt	288 (1,122,783)	25.63 [23.2; 28.26]	234 (1,088,783)	21.46 [19.2; 23.91]
Territorial unit Thuringia	286 (1,082,728)	26.42 [23.9; 29.13]	234 (1,061,232)	22.05 [19.74; 24.57]
Total	6,940 (17,379,066)	39.93 [39.14; 40.73]	5,688 (16,973,138)	33.51 [32.78; 34.25]
