# Supplementary material for: Incidence of Lyme Borreliosis in Germany: Exploring Observed Trends Over Time Using Public Surveillance Data, 2016–2020
Source: Vector Borne Zoonotic Dis. 2023 Apr 12;23(4):237–46. doi: 10.1089/vbz.2022.0046 (PMC10122258; doi:10.1089/vbz.2022.0046)
Supplement: Supplemental data [file Supp_TableS6.pdf]

Table S6. Cases and incidence (per 100,000 person-time, ± 95% CI) of clinically diagnosed and of clinically diagnosed and laboratory confirmed Lyme borreliosis (LB) notified by year and sex in all 9 German States, yearly 2016–2020.

Year	Clinically diagnosed LB				Clinically diagnosed & laboratory confirmed LB			
	Females		Males		Females		Males	
	Cases (population)	Incidence [95% CI]	Cases (population)	Incidence [95% CI]	Cases (population)	Incidence [95% CI]	Cases (population)	Incidence [95% CI]
**2016**	6,062 (17,289,785)	35.06 [34.19;35.95]	5,037 (16,860,614)	29.87 [29.06;30.71]	880 (17,289,785)	5.09 [4.76;5.44]	805 (16,860,614)	4.77 [4.46;5.12]
**2017**	5,874 (17,374,299)	33.81 [32.96;34.68]	4,760 (16,973,973)	28.04 [27.26;28.85]	262 (17,374,299)	1.51 [1.34;1.7]	272 (16,973,973)	1.6 [1.42;1.8]
**2018**	7,051 (17,417,654)	40.48 [39.55;41.44]	5,655 (17,032,865)	33.2 [32.35;34.08]	199 (17,417,654)	1.14 [0.99;1.31]	254 (17,032,865)	1.49 [1.32;1.69]
**2019**	6,547 (17,406,874)	37.61 [36.71;38.53]	5,111 (16,999,031)	30.07 [29.25;30.9]	220 (17,406,874)	1.26 [1.11;1.44]	235 (16,999,031)	1.38 [1.22;1.57]
**2020**	7,524 (17,407,473)	43.22 [42.26;44.21]	6,161 (16,999,394)	36.24 [35.35;37.16]	77 (17,407,473)	0.44 [0.35;0.55]	148 (16,999,394)	0.87 [0.74;1.02]
**Total**	33,058		26,724		1,638		1,714	
