# Supplementary material for: Incidence of Lyme Borreliosis in Germany: Exploring Observed Trends Over Time Using Public Surveillance Data, 2016–2020
Source: Vector Borne Zoonotic Dis. 2023 Apr 12;23(4):237–46. doi: 10.1089/vbz.2022.0046 (PMC10122258; doi:10.1089/vbz.2022.0046)
Supplement: Supplemental data [file Supp_TableS2.pdf]

Table S2. Cases and incidence (per 100,000 person-time, ± 95% CI) of overall Lyme borreliosis notified by year and by 19 German Regions Territorial Units NUTS2, yearly 2016–2020.

		2016		2017		2018		2019		2020	
**State/ NUTS1**	**Region/ NUTS2**	**Cases (population)**	**Incidence [95% CI]**	**Cases (population)**	**Incidence [95% CI]**	**Cases (population)**	**Incidence [95% CI]**	**Cases (population)**	**Incidence [95% CI]**	**Cases (population)**	**Incidence [95% CI]**
Bavaria	Mittelfranken	782 (1,750,224)	44.68 [41.66;47.92]	596 (1,759,669)	33.87 [31.26;36.7]	792 (1,770,228)	44.74 [41.73;47.97]	736 (1,775,205)	41.46 [38.57;44.56]	881 (1,775,136)	49.63 [46.46;53.02]
Bavaria	Niederbayern	988 (1,219,452)	81.02 [76.12;86.23]	702 (1,230,068)	57.07 [53;61.45]	1,135 (1,238,542)	91.64 [86.46;97.13]	924 (1,244,109)	74.27 [69.63;79.21]	1,541 (1,244,147)	123.86 [117.83;130.2]
Bavaria	Oberbayern	777 (4,633,274)	16.77 [15.63;17.99]	798 (4,650,350)	17.16 [16.01;18.39]	1,049 (4,685,127)	22.39 [21.08;23.79]	763 (4,709,877)	16.2 [15.09;17.39]	1,320 (4,710,921)	28.02 [26.55;29.57]
Bavaria	Oberfranken	433 (1,062,316)	40.76 [37.1;44.78]	369 (1,066,782)	34.59 [31.24;38.3]	452 (1,067,548)	42.34 [38.61;46.43]	418 (1,065,240)	39.24 [35.65;43.19]	635 (1,065,436)	59.6 [55.14;64.42]
Bavaria	Oberpfalz	485 (1,098,279)	44.16 [40.4;48.27]	336 (1,104,536)	30.42 [27.34;33.85]	454 (1,109,211)	40.93 [37.33;44.87]	403 (1,112,031)	36.24 [32.87;39.95]	750 (1,112,100)	67.44 [62.78;72.44]
Bavaria	Schwaben	413 (1,857,850)	22.23 [20.19;24.48]	340 (1,873,278)	18.15 [16.32;20.18]	471 (1,887,776)	24.95 [22.8;27.31]	430 (1,899,293)	22.64 [20.6;24.88]	477 (1,899,642)	25.11 [22.96;27.47]
Bavaria	Unterfranken	714 (1,309,131)	54.54 [50.68;58.69]	397 (1,313,265)	30.23 [27.4;33.35]	661 (1,316,995)	50.19 [46.51;54.16]	581 (1,317,759)	44.09 [40.65;47.82]	627 (1,317,504)	47.59 [44.01;51.46]
Berlin	Berlin	900 (3,574,265)	25.18 [23.59;26.88]	763 (3,711,089)	20.56 [19.15;22.07]	755 (3,748,759)	20.14 [18.75;21.63]	851 (3,669,685)	23.19 [21.68;24.8]	943 (3,669,261)	25.7 [24.11;27.39]
Brandenburg	Brandenburg	1,673 (2,494,781)	67.06 [63.92;70.35]	1,743 (2,503,951)	69.61 [66.42;72.95]	1,556 (2,512,108)	61.94 [58.94;65.09]	1,535 (2,521,768)	60.87 [57.9;63.99]	1,608 (2,521,957)	63.76 [60.72;66.95]
Mecklenburg-Vorpommern	Mecklenburg-Vorpommern	973 (1,610,660)	60.41 [56.73;64.33]	1,087 (1,611,086)	67.47 [63.58;71.6]	852 (1,609,673)	52.93 [49.49;56.6]	802 (1,608,181)	49.87 [46.54;53.44]	649 (1,608,028)	40.36 [37.37;43.59]
Rhineland-Palatinate	Koblenz	740 (1,492,236)	49.59 [46.14;53.29]	567 (1,492,891)	37.98 [34.98;41.24]	730 (1,495,902)	48.8 [45.39;52.47]	585 (1,498,079)	39.05 [36.01;42.34]	824 (1,498,182)	55 [51.37;58.88]
Rhineland-Palatinate	Rheinhessen-Pfalz	532 (2,045,367)	26.01 [23.89;28.32]	388 (2,050,740)	18.92 [17.13;20.9]	629 (2,058,246)	30.56 [28.26;33.04]	451 (2,062,186)	21.87 [19.94;23.98]	497 (2,062,241)	24.1 [22.07;26.31]
Rhineland-Palatinate	Trier	199 (528,693)	37.64 [32.76;43.24]	129 (529,557)	24.36 [20.5;28.94]	221 (530,995)	41.62 [36.48;47.48]	149 (533,095)	27.95 [23.81;32.81]	189 (533,145)	35.45 [30.74;40.88]
Saarland	Saarland	197 (996,459)	19.77 [17.2;22.73]	196 (994,419)	19.71 [17.14;22.67]	375 (990,491)	37.86 [34.22;41.89]	288 (986,977)	29.18 [26;32.75]	332 (986,920)	33.64 [30.21;37.46]
Saxony	Chemnitz	905 (1,454,049)	62.24 [58.32;66.43]	767 (1,444,989)	53.08 [49.45;56.97]	907 (1,436,490)	63.14 [59.16;67.38]	1,099 (1,426,347)	77.05 [72.63;81.74]	754 (1,426,409)	52.86 [49.22;56.77]
Saxony	Dresden	987 (1,600,195)	61.68 [57.95;65.65]	940 (1,598,639)	58.8 [55.16;62.68]	1,051 (1,598,236)	65.76 [61.9;69.86]	996 (1,596,666)	62.38 [58.63;66.37]	908 (1,596,624)	56.87 [53.29;60.69]
Saxony	Leipzig	165 (1,027,397)	16.06 [13.79;18.7]	160 (1,037,613)	15.42 [13.21;18]	188 (1,043,285)	18.02 [15.62;20.79]	214 (1,049,020)	20.4 [17.84;23.32]	147 (1,049,251)	14.01 [11.92;16.47]
Saxony-Anhalt	Saxony-Anhalt	506 (2,235,970)	22.63 [20.74;24.69]	573 (2,222,653)	25.78 [23.75;27.98]	530 (2,208,333)	24 [22.04;26.13]	503 (2,194,590)	22.92 [21;25.01]	502 (2,195,015)	22.87 [20.96;24.96]
Thuringia	Thuringia	567 (2,158,356)	26.27 [24.2;28.52]	450 (2,151,052)	20.92 [19.07;22.94]	565 (2,143,399)	26.36 [24.27;28.62]	535 (2,133,174)	25.08 [23.04;27.3]	483 (2,133,392)	22.64 [20.71;24.75]
