# Supplementary material for: Incidence of Lyme Borreliosis in Germany: Exploring Observed Trends Over Time Using Public Surveillance Data, 2016–2020
Source: Vector Borne Zoonotic Dis. 2023 Apr 12;23(4):237–46. doi: 10.1089/vbz.2022.0046 (PMC10122258; doi:10.1089/vbz.2022.0046)
Supplement: Supplemental data [file Supp_TableS3.pdf]

Table S3. Cases and incidence (per 100,000 person-time) of overall Lyme borreliosis by year and by 158 German Counties Territorial Units NUTS3, yearly 2016–2020.

			2016		2017		2018		2019		2020	
State/ NUTS1	Region/ NUTS2	County/ NUTS3	Cases (population)	Incidence [95% CI]	Cases (population)	Incidence [95% CI]	Cases (population)	Incidence [95% CI]	Cases (population)	Incidence [95% CI]	Cases (population)	Incidence [95% CI]
Bavaria	Schwaben	Aichach-Friedberg County	19 (131,397)	14.46 [9.26;22.58]	11 (132,530)	8.3 [4.63;14.86]	37 (133,574)	27.7 [20.1;38.18]	32 (134,680)	23.76 [16.83;33.54]	47 (134,670)	34.9 [26.25;46.4]
Bavaria	Oberbayern	Altötting County	92 (109,420)	84.08 [68.57;103.09]	91 (110,343)	82.47 [67.18;101.23]	95 (111,215)	85.42 [69.89;104.4]	80 (111,514)	71.74 [57.65;89.27]	124 (111,521)	111.19 [93.27;132.54]
Bavaria	Oberpfalz	Amberg-Sulzbach County	76 (103,009)	73.78 [58.96;92.33]	43 (102,846)	41.81 [31.04;56.31]	50 (103,114)	48.49 [36.79;63.92]	38 (103,037)	36.88 [26.87;50.61]	58 (103,056)	56.28 [43.54;72.74]
Bavaria	Mittelfranken	Ansbach County	136 (182,184)	74.65 [63.12;88.29]	123 (183,254)	67.12 [56.26;80.07]	136 (183,958)	73.93 [62.51;87.44]	102 (184,582)	55.26 [45.53;67.07]	127 (184,593)	68.8 [57.83;81.85]
Bavaria	Unterfranken	Aschaffenburg County	95 (173,516)	54.75 [44.79;66.92]	28 (174,021)	16.09 [11.13;23.25]	53 (174,227)	30.42 [23.26;39.78]	38 (174,232)	21.81 [15.89;29.93]	57 (174,205)	32.72 [25.26;42.39]
Bavaria	Schwaben	Augsburg County	89 (247,566)	35.95 [29.22;44.23]	59 (249,788)	23.62 [18.31;30.46]	87 (251,518)	34.59 [28.05;42.66]	108 (253,462)	42.61 [35.3;51.44]	13 (253,411)	5.13 [3;8.78]
Bavaria	Unterfranken	Bad Kissingen County	87 (103,105)	84.38 [68.42;104.06]	50 (103,263)	48.42 [36.73;63.82]	85 (103,218)	82.35 [66.61;101.81]	74 (103,237)	71.68 [57.11;89.97]	103 (103,237)	99.77 [82.28;120.97]
Bavaria	Oberbayern	Bad Tölz-Wolfratshausen County	30 (125,681)	23.87 [16.72;34.07]	21 (126,582)	16.59 [10.85;25.36]	22 (127,241)	17.29 [11.42;26.18]	25 (127,943)	19.54 [13.24;28.84]	36 (127,932)	28.14 [20.33;38.95]
Bavaria	Oberfranken	Bamberg County	22 (146,082)	15.06 [9.95;22.8]	15 (146,628)	10.23 [6.2;16.88]	15 (147,059)	10.2 [6.18;16.83]	30 (147,131)	20.39 [14.28;29.11]	104 (147,163)	70.67 [58.33;85.61]
Bavaria	Oberfranken	Bayreuth County	55 (103,872)	52.95 [40.69;68.91]	23 (103,791)	22.16 [14.77;33.25]	30 (103,663)	28.94 [20.27;41.31]	35 (103,673)	33.76 [24.28;46.95]	52 (103,668)	50.16 [38.26;65.77]
Bavaria	Oberbayern	Berchtesgadener Land County	26 (104,460)	24.89 [16.99;36.47]	28 (105,066)	26.65 [18.44;38.51]	37 (105,714)	35 [25.39;48.24]	30 (105,932)	28.32 [19.84;40.43]	34 (105,919)	32.1 [22.97;44.85]
Bavaria	Oberpfalz	Cham County	92 (126,914)	72.49 [59.12;88.89]	54 (127,328)	42.41 [32.51;55.33]	70 (127,877)	54.74 [43.33;69.15]	66 (128,006)	51.56 [40.53;65.58]	119 (127,998)	92.97 [77.7;111.23]
Bavaria	Oberfranken	Coburg County	40 (86,730)	46.12 [33.87;62.79]	46 (86,891)	52.94 [39.7;70.6]	75 (86,906)	86.3 [68.86;108.15]	62 (86,750)	71.47 [55.76;91.6]	68 (86,746)	78.39 [61.85;99.36]
Bavaria	Oberbayern	Dachau County	4 (150,943)	2.65 [1.03;6.81]	7 (152,838)	4.58 [2.22;9.45]	9 (153,846)	5.85 [3.08;11.12]	8 (155,039)	5.16 [2.61;10.18]	16 (154,889)	10.33 [6.36;16.78]
Bavaria	Niederbayern	Deggendorf County	122 (117,602)	103.74 [86.9;123.84]	86 (118,735)	72.43 [58.66;89.43]	139 (119,324)	116.49 [98.68;137.52]	103 (119,476)	86.21 [71.1;104.53]	153 (119,475)	128.06 [109.32;150.01]
Bavaria	Schwaben	Dillingen a.d.Donau County	39 (94,545)	41.25 [30.18;56.38]	41 (95,150)	43.09 [31.77;58.45]	35 (96,022)	36.45 [26.21;50.69]	56 (96,568)	57.99 [44.66;75.29]	31 (96,573)	32.1 [22.62;45.56]
Bavaria	Niederbayern	Dingolfing-Landau County	70 (95,031)	73.66 [58.31;93.04]	64 (95,837)	66.78 [52.3;85.26]	90 (96,216)	93.54 [76.11;114.95]	65 (96,683)	67.23 [52.76;85.67]	125 (96,682)	129.29 [108.53;154.01]
Bavaria	Schwaben	Donau-Ries County	113 (132,303)	85.41 [71.05;102.67]	71 (133,034)	53.37 [42.32;67.31]	83 (133,505)	62.17 [50.16;77.05]	54 (133,796)	40.36 [30.94;52.65]	105 (133,775)	78.49 [64.85;95]
Bavaria	Oberbayern	Ebersberg County	38 (139,041)	27.33 [19.91;37.51]	67 (140,786)	47.59 [37.48;60.43]	73 (142,134)	51.36 [40.85;64.57]	50 (143,637)	34.81 [26.41;45.89]	79 (143,636)	55 [44.14;68.53]
Bavaria	Oberbayern	Eichstätt County	34 (130,820)	25.99 [18.6;36.31]	21 (131,661)	15.95 [10.43;24.38]	38 (132,358)	28.71 [20.92;39.4]	20 (132,890)	15.05 [9.74;23.25]	57 (132,867)	42.9 [33.12;55.57]
Bavaria	Oberbayern	Erding County	25 (135,428)	18.46 [12.5;27.25]	26 (136,914)	18.99 [12.96;27.82]	37 (137,649)	26.88 [19.5;37.05]	27 (138,178)	19.54 [13.43;28.43]	43 (138,175)	31.12 [23.11;41.91]
Bavaria	Mittelfranken	Erlangen-Höchstadt County	85 (134,643)	63.13 [51.06;78.05]	61 (135,345)	45.07 [35.09;57.88]	79 (136,277)	57.97 [46.52;72.23]	67 (137,267)	48.81 [38.44;61.98]	63 (137,255)	45.9 [35.88;58.72]

Bavaria	Oberfranken	Forchheim County	102 (115,254)	88.5 [72.92;107.41]	46 (115,694)	39.76 [29.81;53.03]	121 (116,101)	104.22 [87.24;124.5]	103 (116,200)	88.64 [73.1;107.48]	203 (116,206)	174.69 [152.27;200.4]
Bavaria	Oberbayern	Freising County	45 (175,781)	25.6 [19.13;34.25]	50 (177,999)	28.09 [21.31;37.03]	40 (179,131)	22.33 [16.4;30.4]	34 (179,989)	18.89 [13.52;26.39]	59 (179,988)	32.78 [25.42;42.28]
Bavaria	Niederbayern	Freyung-Grafenau County	133 (78,180)	170.12 [143.58;201.56]	73 (78,343)	93.18 [74.12;117.13]	167 (78,356)	213.13 [183.19;247.95]	89 (78,359)	113.58 [92.32;139.73]	126 (78,363)	160.79 [135.08;191.39]
Bavaria	Oberbayern	Fürstenfeldbruck County	34 (216,837)	15.68 [11.22;21.91]	18 (217,918)	8.26 [5.23;13.06]	38 (219,273)	17.33 [12.63;23.78]	31 (219,236)	14.14 [9.96;20.07]	63 (219,283)	28.73 [22.46;36.75]
Bavaria	Mittelfranken	Fürth County	110 (115,973)	94.85 [78.71;114.3]	78 (116,192)	67.13 [53.8;83.76]	85 (117,387)	72.41 [58.57;89.52]	96 (117,849)	81.46 [66.72;99.46]	132 (117,857)	112 [94.46;132.79]
Bavaria	Oberbayern	Garmisch-Partenkirchen County	14 (87,884)	15.93 [9.49;26.74]	11 (88,141)	12.48 [6.97;22.35]	16 (88,447)	18.09 [11.14;29.39]	13 (88,435)	14.7 [8.59;25.15]	22 (88,424)	24.88 [16.43;37.67]
Bavaria	Schwaben	Günzburg County	30 (123,508)	24.29 [17.02;34.67]	35 (124,511)	28.11 [20.21;39.09]	59 (125,746)	46.92 [36.38;60.51]	34 (127,008)	26.77 [19.16;37.4]	56 (127,013)	44.09 [33.96;57.24]
Bavaria	Unterfranken	Haßberge County	36 (84,368)	42.67 [30.82;59.06]	32 (84,455)	37.89 [26.84;53.48]	53 (84,597)	62.65 [47.9;81.93]	40 (84,388)	47.4 [34.81;64.53]	45 (84,380)	53.33 [39.86;71.35]
Bavaria	Oberfranken	Hof County	28 (95,923)	29.19 [20.2;42.18]	27 (95,779)	28.19 [19.38;41.01]	23 (95,317)	24.13 [16.08;36.21]	23 (94,806)	24.26 [16.17;36.4]	25 (94,805)	26.37 [17.86;38.93]
Bavaria	Niederbayern	Kelheim County	29 (119,983)	24.17 [16.83;34.71]	15 (121,163)	12.38 [7.5;20.43]	30 (122,249)	24.54 [17.19;35.03]	20 (123,077)	16.25 [10.52;25.1]	49 (123,054)	39.82 [30.12;52.64]
Bavaria	Unterfranken	Kitzingen County	14 (89,744)	15.6 [9.29;26.19]	9 (90,452)	9.95 [5.23;18.91]	59 (90,909)	64.9 [50.32;83.7]	51 (91,153)	55.95 [42.56;73.55]	43 (91,160)	47.17 [35.02;63.53]
Bavaria	Oberfranken	Kronach County	25 (67,604)	36.98 [25.05;54.59]	51 (67,478)	75.58 [57.49;99.35]	43 (67,135)	64.05 [47.56;86.26]	40 (66,745)	59.93 [44.02;81.59]	36 (66,741)	53.94 [38.97;74.66]
Bavaria	Oberfranken	Kulmbach County	51 (71,993)	70.84 [53.89;93.12]	59 (72,013)	81.93 [63.53;105.66]	59 (71,846)	82.12 [63.67;105.9]	45 (71,565)	62.88 [47;84.12]	64 (71,564)	89.43 [70.05;114.17]
Bavaria	Oberbayern	Landsberg a.Lech County	30 (118,718)	25.27 [17.7;36.07]	32 (119,136)	26.86 [19.03;37.91]	39 (120,074)	32.48 [23.76;44.4]	21 (120,275)	17.46 [11.42;26.69]	52 (120,315)	43.22 [32.96;56.67]
Bavaria	Niederbayern	Landshut County	43 (155,459)	27.66 [20.54;37.25]	45 (157,233)	28.62 [21.39;38.29]	86 (158,701)	54.19 [43.89;66.91]	57 (159,888)	35.65 [27.52;46.18]	113 (159,898)	70.67 [58.79;84.95]
Bavaria	Oberfranken	Lichtenfels County	32 (66,639)	48.02 [34.02;67.78]	33 (66,775)	49.42 [35.19;69.39]	14 (66,826)	20.95 [12.48;35.17]	26 (66,769)	38.94 [26.58;57.05]	33 (66,775)	49.42 [35.19;69.39]
Bavaria	Schwaben	Lindau County	1 (80,645)	1.24 [0.22;7.02]	NA (NA)	NA [NA;NA]	5 (81,699)	6.12 [2.61;14.33]	6 (81,967)	7.32 [3.35;15.97]	15 (81,967)	18.3 [11.09;30.19]
Bavaria	Unterfranken	Main-Spessart County	121 (126,305)	95.8 [80.19;114.45]	32 (126,532)	25.29 [17.92;35.7]	84 (126,373)	66.47 [53.7;82.28]	68 (126,160)	53.9 [42.52;68.32]	64 (126,158)	50.73 [39.73;64.77]
Bavaria	Oberbayern	Miesbach County	5 (98,814)	5.06 [2.16;11.85]	5 (99,206)	5.04 [2.15;11.8]	6 (99,668)	6.02 [2.76;13.13]	1 (100,000)	1 [0.18;5.66]	17 (100,000)	17 [10.61;27.23]
Bavaria	Unterfranken	Miltenberg County	44 (128,542)	34.23 [25.5;45.95]	38 (128,465)	29.58 [21.55;40.6]	59 (128,765)	45.82 [35.53;59.09]	48 (128,755)	37.28 [28.12;49.42]	57 (128,755)	44.27 [34.17;57.35]
Bavaria	Oberbayern	Mühldorf a.Inn County	28 (113,223)	24.73 [17.11;35.74]	33 (114,504)	28.82 [20.52;40.47]	35 (115,245)	30.37 [21.84;42.23]	37 (115,878)	31.93 [23.17;44.01]	62 (115,866)	53.51 [41.75;68.58]
Bavaria	Oberbayern	München County	43 (343,450)	12.52 [9.3;16.86]	46 (346,386)	13.28 [9.96;17.71]	59 (348,906)	16.91 [13.11;21.81]	39 (350,404)	11.13 [8.14;15.21]	75 (350,467)	21.4 [17.07;26.82]
Bavaria	Schwaben	Neu-Ulm County	5 (171,233)	2.92 [1.25;6.84]	7 (172,414)	4.06 [1.97;8.38]	13 (174,263)	7.46 [4.36;12.76]	11 (175,159)	6.28 [3.51;11.25]	8 (175,055)	4.57 [2.32;9.02]
Bavaria	Oberbayern	Neuburg-Schrobenhausen County	33 (95,763)	34.46 [24.54;48.39]	23 (96,154)	23.92 [15.94;35.89]	20 (96,665)	20.69 [13.39;31.96]	11 (97,345)	11.3 [6.31;20.24]	11 (97,345)	11.3 [6.31;20.24]
Bavaria	Oberpfalz	Neumarkt i.d.OPf. County	24 (131,651)	18.23 [12.25;27.13]	16 (132,670)	12.06 [7.42;19.59]	18 (133,531)	13.48 [8.53;21.31]	41 (134,559)	30.47 [22.46;41.33]	59 (134,580)	43.84 [33.99;56.54]
Bavaria	Mittelfranken	Neustadt a.d.Aisch-Bad Windsheim County	58 (99,332)	58.39 [45.18;75.47]	60 (99,635)	60.22 [46.79;77.5]	91 (100,364)	90.67 [73.86;111.3]	83 (101,010)	82.17 [66.3;101.84]	94 (101,010)	93.06 [76.06;113.86]
Bavaria	Oberpfalz	Neustadt a.d.Waldnaab County	56 (94,531)	59.24 [45.63;76.91]	34 (94,497)	35.98 [25.75;50.27]	37 (94,364)	39.21 [28.45;54.04]	49 (94,449)	51.88 [39.25;68.57]	71 (94,453)	75.17 [59.6;94.8]
Bavaria	Mittelfranken	Nürnberger Land County	86 (168,892)	50.92 [41.24;62.87]	37 (169,725)	21.8 [15.82;30.05]	74 (170,350)	43.44 [34.61;54.53]	70 (170,773)	40.99 [32.45;51.78]	92 (170,782)	53.87 [43.93;66.06]
Bavaria	Schwaben	Oberallgäu County	15 (153,689)	9.76 [5.92;16.1]	30 (154,560)	19.41 [13.6;27.71]	46 (155,353)	29.61 [22.2;39.49]	55 (156,028)	35.25 [27.09;45.88]	86 (155,995)	55.13 [44.65;68.07]
Bavaria	Schwaben	Ostallgäu County	45 (138,249)	32.55 [24.33;43.55]	42 (139,257)	30.16 [22.32;40.76]	33 (140,306)	23.52 [16.75;33.03]	33 (141,207)	23.37 [16.64;32.82]	66 (141,176)	46.75 [36.75;59.47]

Bavaria	Niederbayern	Passau County	274 (188,900)	145.05 [128.87;163.26]	119 (190,491)	62.47 [52.21;74.74]	201 (192,050)	104.66 [91.16;120.15]	158 (192,659)	82.01 [70.18;95.83]	235 (192,655)	121.98 [107.36;138.59]
Bavaria	Oberbayern	Pfaffenhofen a.d.Ilm County	22 (125,071)	17.59 [11.62;26.63]	29 (126,252)	22.97 [15.99;32.99]	34 (127,150)	26.74 [19.14;37.36]	38 (128,248)	29.63 [21.59;40.66]	55 (128,235)	42.89 [32.96;55.82]
Bavaria	Niederbayern	Regen County	123 (77,189)	159.35 [133.58;190.08]	80 (77,489)	103.24 [82.97;128.46]	172 (77,656)	221.49 [190.8;257.11]	110 (77,410)	142.1 [117.92;171.22]	184 (77,408)	237.7 [205.78;274.56]
Bavaria	Oberpfalz	Regensburg County	80 (190,476)	42 [33.75;52.26]	85 (192,221)	44.22 [35.77;54.67]	161 (193,579)	83.17 [71.28;97.04]	119 (194,064)	61.32 [51.25;73.37]	228 (194,076)	117.48 [103.2;133.74]
Bavaria	Unterfranken	Rhön-Grabfeld County	29 (79,846)	36.32 [25.29;52.16]	19 (79,798)	23.81 [15.24;37.19]	31 (79,692)	38.9 [27.41;55.21]	18 (79,646)	22.6 [14.3;35.72]	35 (79,636)	43.95 [31.6;61.11]
Bavaria	Oberbayern	Rosenheim County	111 (257,481)	43.11 [35.8;51.91]	96 (259,459)	37 [30.3;45.18]	96 (261,011)	36.78 [30.12;44.91]	85 (261,297)	32.53 [26.31;40.22]	123 (261,313)	47.07 [39.46;56.15]
Bavaria	Mittelfranken	Roth County	93 (125,557)	74.07 [60.47;90.72]	73 (126,101)	57.89 [46.05;72.77]	102 (126,960)	80.34 [66.19;97.51]	122 (126,753)	96.25 [80.62;114.9]	124 (126,750)	97.83 [82.07;116.62]
Bavaria	Niederbayern	Rottal-Inn County	95 (119,617)	79.42 [64.98;97.07]	95 (120,375)	78.92 [64.57;96.46]	90 (120,660)	74.59 [60.69;91.67]	140 (121,507)	115.22 [97.66;135.94]	209 (121,505)	172.01 [150.23;196.94]
Bavaria	Oberpfalz	Schwandorf County	93 (145,381)	63.97 [52.23;78.35]	58 (146,502)	39.59 [30.63;51.17]	74 (147,176)	50.28 [40.06;63.11]	58 (147,884)	39.22 [30.34;50.69]	159 (147,866)	107.53 [92.07;125.58]
Bavaria	Unterfranken	Schweinfurt County	63 (114,817)	54.87 [42.89;70.19]	45 (115,119)	39.09 [29.22;52.3]	52 (115,095)	45.18 [34.46;59.24]	52 (115,453)	45.04 [34.35;59.05]	24 (115,440)	20.79 [13.97;30.93]
Bavaria	Oberbayern	Starnberg County	16 (134,680)	11.88 [7.31;19.3]	15 (135,501)	11.07 [6.71;18.27]	50 (136,091)	36.74 [27.87;48.43]	39 (136,650)	28.54 [20.88;39.01]	29 (136,664)	21.22 [14.78;30.47]
Bavaria	Niederbayern	Straubing-Bogen County	69 (99,223)	69.54 [54.96;87.99]	58 (99,845)	58.09 [44.94;75.08]	70 (100,647)	69.55 [55.06;87.85]	95 (101,118)	93.95 [76.87;114.82]	191 (101,122)	188.88 [163.94;217.6]
Bavaria	Oberpfalz	Tirschenreuth County	7 (72,917)	9.6 [4.65;19.82]	14 (72,727)	19.25 [11.47;32.31]	10 (72,516)	13.79 [7.49;25.38]	10 (72,046)	13.88 [7.54;25.55]	9 (72,058)	12.49 [6.57;23.74]
Bavaria	Oberbayern	Traunstein County	36 (175,439)	20.52 [14.82;28.41]	52 (176,271)	29.5 [22.5;38.68]	94 (177,091)	53.08 [43.38;64.95]	35 (177,305)	19.74 [14.19;27.45]	82 (177,336)	46.24 [37.26;57.39]
Bavaria	Schwaben	Unterallgäu County	36 (141,176)	25.5 [18.42;35.3]	24 (142,518)	16.84 [11.32;25.06]	52 (144,044)	36.1 [27.53;47.33]	30 (145,349)	20.64 [14.46;29.46]	24 (145,366)	16.51 [11.1;24.57]
Bavaria	Oberbayern	Weilheim-Schongau County	39 (133,837)	29.14 [21.32;39.83]	35 (134,254)	26.07 [18.75;36.25]	58 (135,356)	42.85 [33.15;55.38]	31 (135,490)	22.88 [16.12;32.47]	83 (135,488)	61.26 [49.43;75.93]
Bavaria	Mittelfranken	Weißenburg-Gunzenhausen County	43 (93,969)	45.76 [33.98;61.63]	30 (94,221)	31.84 [22.3;45.45]	30 (94,399)	31.78 [22.26;45.36]	32 (94,731)	33.78 [23.93;47.68]	55 (94,730)	58.06 [44.61;75.56]
Bavaria	Oberfranken	Wunsiedel i.Fichtelgebirge County	27 (73,250)	36.86 [25.33;53.63]	15 (73,493)	20.41 [12.37;33.67]	14 (73,183)	19.13 [11.4;32.11]	14 (72,652)	19.27 [11.48;32.35]	11 (72,655)	15.14 [8.45;27.11]
Bavaria	Unterfranken	Würzburg County	130 (161,051)	80.72 [67.99;95.83]	125 (161,332)	77.48 [65.04;92.3]	174 (161,830)	107.52 [92.69;124.72]	179 (162,299)	110.29 [95.28;127.66]	145 (162,301)	89.34 [75.94;105.1]
Brandenburg	Brandenburg	Barnim County	189 (179,368)	105.37 [91.39;121.49]	193 (180,864)	106.71 [92.69;122.85]	183 (182,762)	100.13 [86.64;115.72]	207 (185,251)	111.74 [97.53;128.02]	169 (185,246)	91.23 [78.48;106.05]
Brandenburg	Brandenburg	Dahme-Spreewald County	125 (166,069)	75.27 [63.18;89.67]	128 (167,320)	76.5 [64.35;90.94]	123 (169,072)	72.75 [60.98;86.79]	153 (170,797)	89.58 [76.47;104.93]	179 (170,785)	104.81 [90.55;121.32]
Brandenburg	Brandenburg	Elbe-Elster County	36 (104,408)	34.48 [24.91;47.73]	46 (103,464)	44.46 [33.34;59.29]	17 (102,657)	16.56 [10.34;26.52]	17 (101,857)	16.69 [10.42;26.73]	22 (101,805)	21.61 [14.27;32.72]
Brandenburg	Brandenburg	Havelland County	10 (159,744)	6.26 [3.4;11.52]	93 (160,705)	57.87 [47.25;70.88]	84 (161,912)	51.88 [41.91;64.22]	82 (162,989)	50.31 [40.54;62.44]	87 (162,982)	53.38 [43.28;65.83]
Brandenburg	Brandenburg	Märkisch-Oderland County	165 (191,682)	86.08 [73.91;100.25]	161 (192,930)	83.45 [71.52;97.37]	133 (194,331)	68.44 [57.76;81.1]	148 (195,741)	75.61 [64.37;88.81]	85 (195,762)	43.42 [35.12;53.68]
Brandenburg	Brandenburg	Oberhavel County	156 (208,640)	74.77 [63.92;87.45]	159 (209,901)	75.75 [64.86;88.47]	116 (211,255)	54.91 [45.79;65.85]	148 (212,919)	69.51 [59.18;81.64]	176 (212,920)	82.66 [71.32;95.8]
Brandenburg	Brandenburg	Oberspreewald-Lausitz County	34 (111,953)	30.37 [21.73;42.43]	52 (111,111)	46.8 [35.69;61.36]	66 (110,479)	59.74 [46.96;75.99]	52 (109,382)	47.54 [36.26;62.33]	43 (109,359)	39.32 [29.19;52.96]
Brandenburg	Brandenburg	Oder-Spree County	122 (178,859)	68.21 [57.14;81.43]	61 (178,363)	34.2 [26.63;43.92]	101 (178,666)	56.53 [46.53;68.68]	69 (178,803)	38.59 [30.5;48.83]	76 (178,824)	42.5 [33.96;53.19]
Brandenburg	Brandenburg	Ostprignitz-Ruppin County	55 (99,422)	55.32 [42.51;71.99]	34 (99,357)	34.22 [24.49;47.81]	44 (99,077)	44.41 [33.09;59.61]	42 (98,870)	42.48 [31.43;57.41]	38 (98,855)	38.44 [28.01;52.75]
Brandenburg	Brandenburg	Potsdam-Mittelmark County	222 (212,217)	104.61 [91.73;119.3]	241 (213,218)	113.03 [99.64;128.22]	176 (214,660)	81.99 [70.74;95.02]	157 (216,552)	72.5 [62.01;84.76]	150 (216,575)	69.26 [59.03;81.26]
Brandenburg	Brandenburg	Prignitz County	116 (77,811)	149.08 [124.32;178.76]	113 (77,265)	146.25 [121.67;175.79]	82 (76,507)	107.18 [86.36;133.01]	81 (76,156)	106.36 [85.59;132.16]	91 (76,157)	119.49 [97.34;146.67]

Brandenburg	Brandenburg	Spree-Neiße County	47 (116,828)	40.23 [30.26;53.49]	41 (115,460)	35.51 [26.18;48.17]	47 (114,439)	41.07 [30.89;54.61]	33 (113,715)	29.02 [20.67;40.75]	35 (113,710)	30.78 [22.13;42.8]
Brandenburg	Brandenburg	Teltow-Fläming County	104 (164,766)	63.12 [52.1;76.47]	113 (166,888)	67.71 [56.33;81.39]	77 (168,306)	45.75 [36.61;57.17]	71 (169,978)	41.77 [33.12;52.68]	84 (170,006)	49.41 [39.91;61.16]
Brandenburg	Brandenburg	Uckermark County	48 (120,876)	39.71 [29.95;52.64]	54 (120,348)	44.87 [34.39;58.54]	70 (119,556)	58.55 [46.35;73.96]	87 (118,950)	73.14 [59.3;90.2]	134 (118,942)	112.66 [95.14;133.4]
Mecklenburg-Vorpommern	Mecklenburg-Vorpommern	Ludwigslust-Parchim County	259 (212,556)	121.85 [107.89;137.61]	275 (212,519)	129.4 [114.99;145.61]	287 (212,624)	134.98 [120.25;151.51]	160 (211,780)	75.55 [64.72;88.19]	43 (211,823)	20.3 [15.07;27.34]
Mecklenburg-Vorpommern	Mecklenburg-Vorpommern	Mecklenburgische Seenplatte County	82 (261,814)	31.32 [25.24;38.87]	123 (260,593)	47.2 [39.56;56.31]	68 (259,146)	26.24 [20.7;33.26]	58 (258,122)	22.47 [17.38;29.04]	68 (258,065)	26.35 [20.79;33.4]
Mecklenburg-Vorpommern	Mecklenburg-Vorpommern	Nordwestmecklenburg County	191 (156,827)	121.79 [105.71;140.32]	216 (156,988)	137.59 [120.43;157.19]	200 (156,728)	127.61 [111.12;146.55]	190 (157,324)	120.77 [104.78;139.19]	179 (157,321)	113.78 [98.3;131.7]
Mecklenburg-Vorpommern	Mecklenburg-Vorpommern	Rostock County	164 (213,932)	76.66 [65.79;89.32]	157 (214,627)	73.15 [62.57;85.52]	96 (215,102)	44.63 [36.55;54.49]	109 (215,799)	50.51 [41.88;60.92]	160 (215,808)	74.14 [63.51;86.55]
Mecklenburg-Vorpommern	Mecklenburg-Vorpommern	Vorpommern-Greifswald County	54 (237,363)	22.75 [17.44;29.68]	74 (237,103)	31.21 [24.86;39.18]	39 (236,650)	16.48 [12.06;22.53]	62 (235,652)	26.31 [20.53;33.72]	80 (235,641)	33.95 [27.28;42.25]
Mecklenburg-Vorpommern	Mecklenburg-Vorpommern	Vorpommern-Rügen County	59 (224,933)	26.23 [20.34;33.83]	88 (225,122)	39.09 [31.73;48.15]	68 (224,719)	30.26 [23.87;38.36]	88 (224,719)	39.16 [31.79;48.24]	70 (224,719)	31.15 [24.66;39.35]
Rhineland-Palatinate	Koblenz	Ahrweiler County	47 (128,450)	36.59 [27.52;48.65]	41 (128,931)	31.8 [23.44;43.13]	31 (129,707)	23.9 [16.84;33.92]	32 (130,081)	24.6 [17.43;34.72]	44 (130,101)	33.82 [25.2;45.39]
Rhineland-Palatinate	Koblenz	Altenkirchen County	60 (128,949)	46.53 [36.15;59.88]	69 (128,779)	53.58 [42.34;67.8]	170 (128,700)	132.09 [113.68;153.48]	121 (128,806)	93.94 [78.63;112.22]	161 (128,800)	125 [107.13;145.84]
Rhineland-Palatinate	Rheinhessen-Pfalz	Alzey-Worms County	17 (128,205)	13.26 [8.28;21.24]	10 (128,535)	7.78 [4.23;14.32]	17 (129,278)	13.15 [8.21;21.06]	7 (129,630)	5.4 [2.62;11.15]	6 (129,590)	4.63 [2.12;10.1]
Rhineland-Palatinate	Rheinhessen-Pfalz	Bad Dürkheim County	51 (132,951)	38.36 [29.18;50.43]	39 (132,743)	29.38 [21.49;40.16]	59 (132,674)	44.47 [34.48;57.35]	44 (132,690)	33.16 [24.7;44.51]	44 (132,690)	33.16 [24.7;44.51]
Rhineland-Palatinate	Koblenz	Bad Kreuznach County	142 (157,393)	90.22 [76.56;106.32]	107 (157,538)	67.92 [56.22;82.06]	81 (158,080)	51.24 [41.23;63.68]	131 (158,346)	82.73 [69.73;98.15]	141 (158,338)	89.05 [75.52;105]
Rhineland-Palatinate	Trier	Bernkastel-Wittlich County	32 (112,006)	28.57 [20.24;40.33]	22 (112,130)	19.62 [12.96;29.71]	14 (112,269)	12.47 [7.43;20.93]	10 (112,486)	8.89 [4.83;16.37]	15 (112,444)	13.34 [8.08;22.01]
Rhineland-Palatinate	Koblenz	Birkenfeld County	73 (80,797)	90.35 [71.87;113.57]	64 (80,727)	79.28 [62.1;101.22]	62 (80,719)	76.81 [59.93;98.44]	54 (80,947)	66.71 [51.14;87.02]	95 (80,954)	117.35 [96.02;143.42]
Rhineland-Palatinate	Trier	Bitburg-Prüm County	46 (98,018)	46.93 [35.19;62.59]	26 (98,224)	26.47 [18.07;38.78]	73 (98,555)	74.07 [58.92;93.11]	39 (99,060)	39.37 [28.8;53.81]	38 (99,062)	38.36 [27.95;52.64]
Rhineland-Palatinate	Koblenz	Cochem-Zell County	50 (61,866)	80.82 [61.31;106.52]	18 (61,665)	29.19 [18.47;46.14]	50 (61,584)	81.19 [61.59;107.01]	20 (61,369)	32.59 [21.1;50.34]	65 (61,373)	105.91 [83.11;134.95]
Rhineland-Palatinate	Rheinhessen-Pfalz	Donnersbergkreis County	74 (75,203)	98.4 [78.4;123.5]	45 (75,100)	59.92 [44.79;80.16]	69 (75,098)	91.88 [72.61;116.25]	38 (75,322)	50.45 [36.76;69.23]	43 (75,320)	57.09 [42.39;76.88]
Rhineland-Palatinate	Rheinhessen-Pfalz	Germersheim County	18 (128,205)	14.04 [8.88;22.19]	13 (128,458)	10.12 [5.91;17.32]	17 (129,081)	13.17 [8.22;21.09]	21 (128,993)	16.28 [10.65;24.89]	8 (129,032)	6.2 [3.14;12.23]
Rhineland-Palatinate	Rheinhessen-Pfalz	Kaiserslautern County	40 (105,513)	37.91 [27.84;51.61]	37 (105,654)	35.02 [25.41;48.26]	61 (106,050)	57.52 [44.79;73.87]	50 (105,977)	47.18 [35.79;62.19]	63 (105,971)	59.45 [46.47;76.05]
Rhineland-Palatinate	Rheinhessen-Pfalz	Kusel County	47 (70,901)	66.29 [49.86;88.13]	25 (70,761)	35.33 [23.93;52.15]	50 (70,522)	70.9 [53.79;93.45]	48 (70,217)	68.36 [51.57;90.62]	49 (70,221)	69.78 [52.79;92.23]
Rhineland-Palatinate	Rheinhessen-Pfalz	Mainz-Bingen County	34 (209,231)	16.25 [11.63;22.71]	14 (209,895)	6.67 [3.97;11.2]	28 (210,843)	13.28 [9.19;19.19]	8 (211,640)	3.78 [1.92;7.46]	25 (211,506)	11.82 [8.01;17.45]
Rhineland-Palatinate	Koblenz	Mayen-Koblenz County	55 (212,931)	25.83 [19.85;33.62]	27 (213,608)	12.64 [8.69;18.39]	62 (214,236)	28.94 [22.58;37.09]	36 (214,413)	16.79 [12.13;23.24]	44 (214,425)	20.52 [15.29;27.54]
Rhineland-Palatinate	Koblenz	Neuwied County	52 (181,564)	28.64 [21.84;37.55]	47 (181,678)	25.87 [19.46;34.4]	69 (181,962)	37.92 [29.97;47.98]	49 (182,836)	26.8 [20.27;35.43]	52 (182,841)	28.44 [21.69;37.29]
Rhineland-Palatinate	Koblenz	Rhein-Hunsrück-Kreis County	73 (103,020)	70.86 [56.37;89.08]	49 (102,941)	47.6 [36.01;62.92]	44 (102,948)	42.74 [31.84;57.37]	32 (103,159)	31.02 [21.97;43.79]	62 (103,161)	60.1 [46.89;77.03]
Rhineland-Palatinate	Koblenz	Rhein-Lahn-Kreis County	104 (122,555)	84.86 [70.05;102.8]	74 (122,375)	60.47 [48.18;75.9]	77 (122,300)	62.96 [50.38;78.67]	45 (122,283)	36.8 [27.51;49.23]	57 (122,291)	46.61 [35.98;60.38]
Rhineland-Palatinate	Rheinhessen-Pfalz	Rhein-Pfalz-Kreis County	70 (153,039)	45.74 [36.21;57.78]	20 (153,610)	13.02 [8.43;20.11]	24 (154,242)	15.56 [10.46;23.15]	26 (154,578)	16.82 [11.48;24.65]	10 (154,560)	6.47 [3.51;11.91]
Rhineland-Palatinate	Rheinhessen-Pfalz	Südliche Weinstraße County	47 (110,875)	42.39 [31.88;56.36]	64 (110,631)	57.85 [45.31;73.86]	66 (110,349)	59.81 [47.02;76.08]	56 (110,519)	50.67 [39.03;65.79]	45 (110,511)	40.72 [30.44;54.48]
Rhineland-Palatinate	Rheinhessen-Pfalz	Südwestpfalz County	51 (95,955)	53.15 [40.43;69.87]	33 (95,486)	34.56 [24.61;48.53]	62 (95,107)	65.19 [50.86;83.55]	29 (94,833)	30.58 [21.29;43.91]	52 (94,839)	54.83 [41.82;71.89]

Rhineland-Palatinate	Trier	Trier-Saarburg County	26 (147,811)	17.59 [12;25.77]	26 (148,571)	17.5 [11.94;25.64]	37 (148,953)	24.84 [18.02;34.23]	36 (149,378)	24.1 [17.41;33.36]	40 (149,421)	26.77 [19.66;36.45]
Rhineland-Palatinate	Trier	Vulkaneifel County	82 (60,759)	134.96 [108.75;167.47]	51 (60,707)	84.01 [63.91;110.43]	88 (60,602)	145.21 [117.89;178.85]	52 (60,648)	85.74 [65.39;112.41]	86 (60,645)	141.81 [114.85;175.08]
Rhineland-Palatinate	Koblenz	Westerwaldkreis County	78 (201,031)	38.8 [31.09;48.42]	70 (201,034)	34.82 [27.56;43.99]	80 (201,613)	39.68 [31.89;49.38]	61 (201,920)	30.21 [23.52;38.8]	97 (201,915)	48.04 [39.39;58.6]
Saarland	Saarland	Merzig-Wadern County	21 (103,909)	20.21 [13.22;30.9]	22 (103,676)	21.22 [14.01;32.13]	20 (103,359)	19.35 [12.53;29.89]	31 (103,230)	30.03 [21.16;42.62]	25 (103,263)	24.21 [16.4;35.74]
Saarland	Saarland	Neunkirchen County	22 (133,983)	16.42 [10.84;24.86]	17 (133,333)	12.75 [7.96;20.42]	31 (132,196)	23.45 [16.52;33.28]	32 (131,417)	24.35 [17.25;34.37]	4 (131,579)	3.04 [1.18;7.82]
Saarland	Saarland	Saar-Pfalz-Kreis County	33 (144,105)	22.9 [16.31;32.16]	35 (143,384)	24.41 [17.55;33.94]	68 (142,617)	47.68 [37.62;60.43]	39 (142,180)	27.43 [20.07;37.49]	51 (142,180)	35.87 [27.29;47.15]
Saarland	Saarland	Saarlouis County	13 (196,672)	6.61 [3.86;11.31]	34 (195,853)	17.36 [12.42;24.26]	71 (195,216)	36.37 [28.84;45.87]	72 (194,332)	37.05 [29.42;46.65]	83 (194,334)	42.71 [34.46;52.94]
Saarland	Saarland	Sankt Wendel County	48 (88,463)	54.26 [40.93;71.93]	46 (87,870)	52.35 [39.25;69.81]	102 (87,396)	116.71 [96.16;141.64]	79 (87,004)	90.8 [72.87;113.14]	107 (87,006)	122.98 [101.79;148.57]
Saarland	Saarland	Stadtverband Saarbrücken County	60 (329,670)	18.2 [14.14;23.42]	42 (330,189)	12.72 [9.41;17.19]	83 (329,758)	25.17 [20.31;31.2]	35 (328,638)	10.65 [7.66;14.81]	62 (328,738)	18.86 [14.71;24.17]
Saxony	Dresden	Bautzen County	116 (304,702)	38.07 [31.74;45.65]	128 (302,600)	42.3 [35.58;50.29]	140 (300,881)	46.53 [39.44;54.9]	100 (299,760)	33.36 [27.43;40.57]	74 (299,716)	24.69 [19.67;30.99]
Saxony	Chemnitz	Erzgebirgskreis County	262 (344,148)	76.13 [67.46;85.92]	219 (340,379)	64.34 [56.37;73.44]	236 (337,673)	69.89 [61.53;79.39]	337 (334,957)	100.61 [90.43;111.93]	247 (334,961)	73.74 [65.1;83.52]
Saxony	Leipzig	Leipzig County	70 (258,303)	27.1 [21.45;34.23]	72 (257,972)	27.91 [22.17;35.14]	84 (257,748)	32.59 [26.33;40.34]	104 (258,129)	40.29 [33.26;48.81]	83 (258,165)	32.15 [25.94;39.85]
Saxony	Dresden	Meißen County	157 (243,902)	64.37 [55.06;75.25]	155 (242,871)	63.82 [54.54;74.68]	137 (242,178)	56.57 [47.86;66.87]	147 (241,737)	60.81 [51.74;71.46]	109 (241,739)	45.09 [37.38;54.38]
Saxony	Chemnitz	Mittelsachsen County	223 (310,498)	71.82 [62.99;81.88]	195 (308,154)	63.28 [55;72.8]	241 (306,187)	78.71 [69.38;89.29]	238 (304,114)	78.26 [68.93;88.85]	163 (304,104)	53.6 [45.98;62.48]
Saxony	Leipzig	Nordsachsen County	30 (198,020)	15.15 [10.61;21.63]	37 (197,755)	18.71 [13.58;25.79]	42 (197,647)	21.25 [15.72;28.72]	35 (197,740)	17.7 [12.73;24.61]	24 (197,694)	12.14 [8.16;18.06]
Saxony	Dresden	Sächsische Schweiz-Osterzgebirge County	340 (246,074)	138.17 [124.25;153.65]	288 (245,420)	117.35 [104.56;131.7]	307 (245,620)	124.99 [111.78;139.76]	279 (245,577)	113.61 [101.04;127.74]	309 (245,589)	125.82 [112.56;140.64]
Saxony	Chemnitz	Vogtlandkreis County	191 (231,039)	82.67 [71.75;95.25]	174 (229,582)	75.79 [65.34;87.91]	220 (227,790)	96.58 [84.64;110.21]	263 (226,003)	116.37 [103.14;131.3]	183 (226,010)	80.97 [70.06;93.58]
Saxony	Chemnitz	Zwickau County	63 (322,086)	19.56 [15.29;25.02]	59 (319,957)	18.44 [14.3;23.78]	51 (317,559)	16.06 [12.22;21.11]	79 (314,992)	25.08 [20.13;31.25]	88 (314,961)	27.94 [22.68;34.42]
Saxony-Anhalt	Saxony-Anhalt	Altmarkkreis Salzwedel County	11 (85,205)	12.91 [7.21;23.12]	12 (84,448)	14.21 [8.13;24.84]	20 (83,752)	23.88 [15.46;36.88]	18 (83,179)	21.64 [13.69;34.21]	16 (83,160)	19.24 [11.84;31.25]
Saxony-Anhalt	Saxony-Anhalt	Anhalt-Bitterfeld County	6 (163,043)	3.68 [1.69;8.03]	11 (161,290)	6.82 [3.81;12.21]	3 (159,574)	1.88 [0.64;5.53]	8 (158,416)	5.05 [2.56;9.97]	13 (158,537)	8.2 [4.79;14.03]
Saxony-Anhalt	Saxony-Anhalt	Börde County	110 (172,956)	63.6 [52.78;76.64]	125 (172,628)	72.41 [60.78;86.26]	135 (171,734)	78.61 [66.42;93.03]	127 (170,929)	74.3 [62.46;88.39]	116 (170,915)	67.87 [56.59;81.39]
Saxony-Anhalt	Saxony-Anhalt	Burgenlandkreis County	6 (182,927)	3.28 [1.5;7.16]	7 (181,818)	3.85 [1.86;7.95]	5 (180,505)	2.77 [1.18;6.48]	6 (179,104)	3.35 [1.54;7.31]	2 (178,571)	1.12 [0.31;4.08]
Saxony-Anhalt	Saxony-Anhalt	Harz County	4 (219,780)	1.82 [0.71;4.68]	24 (216,216)	11.1 [7.46;16.52]	28 (214,395)	13.06 [9.04;18.87]	15 (213,371)	7.03 [4.26;11.6]	11 (213,178)	5.16 [2.88;9.24]
Saxony-Anhalt	Saxony-Anhalt	Jerichower Land County	45 (91,352)	49.26 [36.82;65.9]	31 (90,458)	34.27 [24.15;48.64]	39 (89,924)	43.37 [31.73;59.28]	48 (89,586)	53.58 [40.42;71.03]	48 (89,586)	53.58 [40.42;71.03]
Saxony-Anhalt	Saxony-Anhalt	Mansfeld-Südharz County	46 (139,775)	32.91 [24.68;43.89]	37 (138,008)	26.81 [19.45;36.95]	20 (136,240)	14.68 [9.5;22.68]	31 (134,959)	22.97 [16.18;32.6]	49 (134,949)	36.31 [27.47;48]
Saxony-Anhalt	Saxony-Anhalt	Saalekreis County	45 (185,950)	24.2 [18.09;32.38]	41 (185,520)	22.1 [16.29;29.98]	18 (184,615)	9.75 [6.17;15.41]	24 (183,767)	13.06 [8.78;19.43]	16 (183,908)	8.7 [5.36;14.13]
Saxony-Anhalt	Saxony-Anhalt	Salzlandkreis County	97 (194,584)	49.85 [40.87;60.8]	117 (192,751)	60.7 [50.65;72.74]	118 (190,568)	61.92 [51.71;74.14]	107 (189,113)	56.58 [46.83;68.36]	141 (189,135)	74.55 [63.22;87.91]
Saxony-Anhalt	Saxony-Anhalt	Stendal County	15 (114,416)	13.11 [7.95;21.63]	25 (113,173)	22.09 [14.96;32.61]	26 (111,972)	23.22 [15.85;34.02]	15 (111,193)	13.49 [8.18;22.26]	25 (111,210)	22.48 [15.23;33.18]
Saxony-Anhalt	Saxony-Anhalt	Wittenberg County	18 (127,479)	14.12 [8.93;22.32]	11 (126,874)	8.67 [4.84;15.53]	13 (125,847)	10.33 [6.04;17.67]	9 (125,000)	7.2 [3.79;13.68]	6 (125,000)	4.8 [2.2;10.47]
Thuringia	Thuringia	Altenburger Land County	13 (91,614)	14.19 [8.29;24.28]	8 (90,600)	8.83 [4.47;17.42]	18 (90,135)	19.97 [12.63;31.57]	16 (89,385)	17.9 [11.02;29.08]	18 (89,374)	20.14 [12.74;31.84]

Thuringia	Thuringia	Eichsfeld County	4 (101,010)	3.96 [1.54;10.18]	3 (100,671)	2.98 [1.01;8.76]	3 (100,334)	2.99 [1.02;8.79]	NA (NA)	NA [NA;NA]	NA (NA)	NA [NA;NA]
Thuringia	Thuringia	Gotha County	26 (135,417)	19.2 [13.1;28.13]	24 (135,517)	17.71 [11.9;26.35]	35 (135,449)	25.84 [18.58;35.93]	17 (134,921)	12.6 [7.87;20.18]	16 (134,907)	11.86 [7.3;19.27]
Thuringia	Thuringia	Greiz County	24 (99,709)	24.07 [16.18;35.81]	25 (99,285)	25.18 [17.06;37.17]	24 (98,160)	24.45 [16.43;36.38]	27 (97,403)	27.72 [19.05;40.33]	24 (97,403)	24.64 [16.56;36.66]
Thuringia	Thuringia	Hildburghausen County	23 (64,336)	35.75 [23.82;53.64]	30 (63,925)	46.93 [32.88;66.99]	22 (63,547)	34.62 [22.86;52.42]	30 (63,198)	47.47 [33.25;67.76]	27 (63,202)	42.72 [29.36;62.15]
Thuringia	Thuringia	Ilm-Kreis County	72 (109,174)	65.95 [52.38;83.04]	43 (108,833)	39.51 [29.34;53.21]	68 (108,748)	62.53 [49.33;79.26]	53 (106,255)	49.88 [38.14;65.23]	41 (106,245)	38.59 [28.45;52.34]
Thuringia	Thuringia	Kyffhäuserkreis County	6 (76,726)	7.82 [3.58;17.06]	3 (75,758)	3.96 [1.35;11.64]	2 (74,906)	2.67 [0.73;9.74]	1 (74,074)	1.35 [0.24;7.65]	1 (74,074)	1.35 [0.24;7.65]
Thuringia	Thuringia	Nordhausen County	33 (85,095)	38.78 [27.62;54.45]	36 (84,706)	42.5 [30.7;58.83]	33 (83,820)	39.37 [28.04;55.28]	16 (83,420)	19.18 [11.81;31.16]	12 (83,391)	14.39 [8.23;25.15]
Thuringia	Thuringia	Saale-Holzland-Kreis County	23 (84,528)	27.21 [18.13;40.83]	17 (83,008)	20.48 [12.79;32.8]	27 (83,051)	32.51 [22.34;47.3]	23 (82,943)	27.73 [18.48;41.61]	20 (82,953)	24.11 [15.61;37.24]
Thuringia	Thuringia	Saale-Orla-Kreis County	30 (82,372)	36.42 [25.51;51.99]	25 (81,513)	30.67 [20.78;45.27]	56 (80,866)	69.25 [53.34;89.91]	50 (80,308)	62.26 [47.23;82.06]	28 (80,321)	34.86 [24.12;50.38]
Thuringia	Thuringia	Saalfeld-Rudolstadt County	86 (108,312)	79.4 [64.3;98.04]	72 (107,367)	67.06 [53.26;84.43]	74 (106,352)	69.58 [55.43;87.33]	62 (103,196)	60.08 [46.87;77]	84 (103,194)	81.4 [65.76;100.76]
Thuringia	Thuringia	Schmalkalden-Meiningen County	30 (123,508)	24.29 [17.02;34.67]	23 (122,929)	18.71 [12.47;28.08]	31 (122,336)	25.34 [17.85;35.97]	35 (124,911)	28.02 [20.15;38.96]	41 (124,924)	32.82 [24.2;44.52]
Thuringia	Thuringia	Sömmerda County	10 (70,126)	14.26 [7.75;26.25]	4 (70,053)	5.71 [2.22;14.68]	4 (69,686)	5.74 [2.23;14.76]	3 (69,444)	4.32 [1.47;12.7]	1 (69,444)	1.44 [0.25;8.16]
Thuringia	Thuringia	Sonneberg County	18 (56,515)	31.85 [20.15;50.34]	22 (56,367)	39.03 [25.78;59.09]	28 (56,191)	49.83 [34.48;72.01]	29 (57,711)	50.25 [34.99;72.16]	21 (57,724)	36.38 [23.8;55.61]
Thuringia	Thuringia	Unstrut-Hainich-Kreis County	4 (103,896)	3.85 [1.5;9.9]	11 (103,481)	10.63 [5.94;19.04]	7 (102,941)	6.8 [3.29;14.04]	7 (102,190)	6.85 [3.32;14.14]	11 (102,230)	10.76 [6.01;19.27]
Thuringia	Thuringia	Wartburgkreis County	22 (124,717)	17.64 [11.65;26.71]	13 (123,810)	10.5 [6.14;17.97]	13 (122,990)	10.57 [6.18;18.09]	10 (118,906)	8.41 [4.57;15.48]	13 (118,939)	10.93 [6.39;18.7]
Thuringia	Thuringia	Weimarer Land County	18 (82,305)	21.87 [13.83;34.57]	6 (82,079)	7.31 [3.35;15.95]	3 (81,967)	3.66 [1.24;10.76]	4 (82,136)	4.87 [1.89;12.52]	7 (82,160)	8.52 [4.13;17.59]
